# Generation of marmoset monkeys with a non-mosaic disruption of the *OTOF* gene as a model of human deafness

**DOI:** 10.1038/s41467-026-71047-1

**Published:** 2026-03-28

**Authors:** Tobias Kahland, Dimitri Leonid Lindenwald, Marcus Jeschke, Kathrin Kusch, Olena Tkachenko Eikel, Mara Uhl, Nancy Rüger, Charis Drummer, Bettina Wolf, Fritz Benseler, Nils Brose, Rüdiger Behr, Tobias Moser

**Affiliations:** 1 Platform Stem Cell Biology and Regeneration, German Primate Center—Leibniz Institute for Primate Research, Göttingen, Germany; 2 Institute for Auditory Neuroscience and InnerEarLab, University Medical Center Göttingen, Göttingen, Germany; 3 Cognitive Hearing in Primates group, Auditory Neuroscience and Optogenetics Laboratory, German Primate Center, Göttingen, Germany; 4 Else Kröner Fresenius Center for Optogenetic Therapies, University Medical Center, Göttingen, Germany; 5 Collaborative Research Center CRC 1690 “Disease Mechanisms and Functional Restoration of Sensory and Motor Systems”, University of Göttingen, Göttingen, Germany; 6 Functional Auditory Genomics group, Auditory Neuroscience and Optogenetics Laboratory, German Primate Center, Göttingen, Germany; 7 Cluster of Excellence “Multiscale Bioimaging: from Molecular Machines to Networks of Excitable Cells” (MBExC), University of Göttingen, Göttingen, Germany; 8 Department of Molecular Neurobiology, Max Planck Institute for Multidisciplinary Sciences, Göttingen, Germany; 9 Auditory Neuroscience and Optogenetics Laboratory, German Primate Center, Göttingen, Germany; 10 Auditory Neuroscience and Synaptic Nanophysiology Group, Max-Planck-Institute for Multidisciplinary Sciences, Göttingen, Germany

**Keywords:** Diseases of the nervous system, CRISPR-Cas systems

## Abstract

Disabling hearing impairment is a common human sensory deficit. *OTOF* is a major deafness gene. It codes for the synaptic protein otoferlin and is essential for transmitter release by inner hair cells (IHCs). Upon genetic loss of otoferlin, cochlear structure and function remain intact up to the IHC synapses, which fail to encode sound. Building on preclinical hearing restoration by AAV-mediated cochlear gene transfer in mice, clinical *OTOF-*gene-therapy trials are now targeting the pediatric population. However, preclinical optimization and characterization remain urgent needs for the development of *OTOF-*gene-therapy. Here, we report on the generation and characterization of a marmoset KO that models *OTOF*-related auditory synaptopathy and can thus address these needs. Following ovary stimulation, harvesting, in vitro maturation and fertilization of oocytes, we injected the zygotes with Cas9 and guide RNAs to disrupt *OTOF*. Mutant embryos were transferred into the uterus of foster mothers. Marmosets with biallelic, non-mosaic *OTOF*-KO were normally born and raised by their respective foster parents. Auditory brainstem recordings and otoacoustic emissions revealed profound auditory synaptopathy and *OTOF*-KO was further validated by the lack of otoferlin expression in IHCs. The new non-human primate model of *OTOF*-related auditory synaptopathy will serve studies of specificity, efficacy, and longevity of novel inner ear therapies.

## Abstract (web summary)

Advancing our understanding of hereditary hearing loss and the development of gene therapies requires disease models in non-human primates. Here, the authors report on the generation and characterization of a non-mosaic marmoset model of OTOF-related auditory synaptopathy.

## Introduction

Following decades of highly informative disease modeling in mice^1–3^, efforts have been undertaken to develop primate models. This is motivated by the facts that ~1% of human and mouse genes lack an orthologue in the respective other species, and some 1:1 human:mouse orthologs have evolved new temporal and spatial expression trajectories, so that corresponding gene variants can cause different phenotypes and disease patterns in the two species^4–6^. Indeed, while many forms of monogenic human deafness, including *OTOF*-related auditory synaptopathy, can be modeled in mice there are notable exceptions, e.g. in the case of the deafness genes *GJB3, CRYM, GRHL2, DFNA5*, and *ATP6B1*^6^. Disease modeling based on human pluripotent stem cells partially solves these problems. Cells or tissue models derived from patient-specific induced pluripotent stem cells (hiPSCs) or genetically modified wild-type hiPSCs can serve the investigation of disease mechanisms and the development of therapeutic approaches^7,8^. However, even with the help of rapidly developing 2D and 3D cell culture models and organoid technology, it is not yet possible to generate fully differentiated and functionally mature tissues or organs in vitro, let alone distributed systems such as the auditory system that is in the focus of the present study. Hence, the clinical translation of *OTOF-*gene-therapy has relied on safety and efficacy studies in *Otof*-KO mice and safety studies in wild-type non-human primates (NHPs)^9^. Further work, ideally including studies in an NHP model, is required to evaluate the efficacy, specificity and longevity of the gene therapy^9^. For example, whether expression of transgenic otoferlin in the *OTOF-*deficient primate cochlea is limited to inner hair cells (IHCs, i.e. target-cell specific expression) is one key open question that can only be addressed by post-mortem histology. Genetic modification of NHPs can close this gap between mouse models and hiPSC-derived models on the one hand and clinical trials on the other. Moreover, genetically modified NHPs will enable in-depth biomedical research^10^.

NHPs are evolutionarily much closer to humans than rodents: genetic studies have shown that humans share approximately 99% of their DNA with chimpanzees^11^, our closest relatives, while sharing ~90% with mice^12^. This is also reflected in a more similar development, anatomy and physiology^10^. For example, humans and NHPs start hearing in utero (onset of hearing in marmosets likely in last month before birth^6^), while rodents show a postnatal hearing onset. This is particularly relevant in the context of gene therapy studies, in which postnatal intervention could meet a structurally intact sensory organ in rodents, while degeneration e.g. of dysfunctional hair cells might limit the feasibility of gene therapy in NHPs and humans^9^. Human canonical OTOF and the long marmoset OTOF isoform (UniProt F7IDY5) are both 1997 aa long. The functionally critical domain architecture (eight C2 domains, C-terminal TM helix) is conserved across mammals, and the sequences of human and marmoset OTOF are over 90% identical, even more similar in the C2 domain cores, and almost completely conserved in the TM region^13^. A recent bioinformatics study found that marmosets have a large number of genes containing human disease-associated single-nucleotide polymorphisms^14^. Therefore, NHPs, such as marmosets, are used with great success in biomedicine, particularly in neurology, immunology and infectious diseases, in the development of therapeutic monoclonal antibodies and gene therapies, in translational stem cell, developmental and regenerative biology as well as in systemic and preclinical neuroscience^10,15^. Understanding the neurobiological basis of vocal communication in primates, such as in marmosets with highly complex social communication^16^, for an example, would benefit from NHPs with altered auditory function^16,17^.

Marmosets are small NHPs and belong to the Platyrrhini (New World monkeys). Their small body size and weight (between 250 and 500 grams) allow them to be handled without sedation or anesthesia, which is important in terms of animal welfare^18^. Despite their small body size, marmosets have typical primate characteristics, including, e.g., forward-facing eyes and a *fovea centralis* of the retina^19^. Moreover, marmosets have properties that make them particularly suitable for studies that require efficient breeding or involve transgenerational aspects. Marmosets typically have 2–3 offspring twice a year, with a gestation period of 145 days^20^. The generation time for marmosets is 1.5-2 years. Macaques, on the other hand, typically give birth to a single offspring every 1–2 years and the generation time is ~4–6 years^20^. Marmosets can therefore reproduce faster than macaques through natural mating, making the expansion of a single breeding pair with specific characteristics more efficient. However, despite the significantly different generation times and fecundities of macaques and marmosets, the generation times of NHPs are generally much longer than those of rodents and also longer than in other large animal models such as pigs (about 1 year). Considering animal welfare, research project management and the high costs of keeping NHPs, avoiding (i) genetic mosaicism and (ii) off-target modifications is of particular importance in the generation of genetically modified NHPs. Both can cause severe problems in the genetic characterization, phenotyping and breeding, and can therefore seriously confound scientific and clinical progress.

Capitalizing on recent progress in gene editing and reproductive biology^21,22^, we targeted the *OTOF* gene to generate a marmoset model of human hearing impairment (HI). Our work complements prior preliminary efforts to generate an NHP model of Usher syndrome in rhesus macaques^23^. We consider this ethically justified as HI is common and substantially reduces the quality of life of affected individuals. One to two per thousand children are born with disabling HI, which in over 50% is caused by defects in individual genes (monogenic deafness) of which at least 150 have been identified so far (http://hereditaryhearingloss.org/). Deaf children do not develop vocal speech unless provided with a cochlear implant that bypasses the dysfunctional or lost sensory hair cells and electrically stimulates the auditory nerve. While this approach partially restores hearing in most deaf children, it has substantial shortcomings such as limited understanding of speech in background noise. So there remains a major unmet medical need for improved hearing restoration^24^. However, so far, despite major research efforts, a causal treatment based on pharmacology, gene therapy, or stem cells has not been clinically available. This is now changing for *OTOF*-related auditory synaptopathy. *OTOF* mutations cause autosomal recessive deafness DFNB9^25^. HI due to *OTOF* mutations is prevalent (up to 7% of hereditary non-syndromic HI) and ranges from mild threshold increases (some with temperature-dependent worsening) to deafness^26–28^.

*OTOF*-related auditory synaptopathy results from defective sound encoding at the synapse between inner hair cells (IHCs) and auditory nerve fibers: auditory synaptopathy (review in ref. ^27^). Otoferlin has multiple functions in Ca^2+^ triggered release of synaptic vesicles (SVs), in coupling of exo- and endocytosis, and in replenishment of SVs^13,29–33^. Except for the early loss of synapses (approximately 50% in mouse *Otof*-KO), cochlear structure and sensory hair cells in particular are well maintained^29,34^. In fact, outer hair cell function, probed as cochlear microphonic potentials and otoacoustic emissions, can remain over years in *OTOF* mutant deaf ears^26,35^. This offers a therapeutic window for gene therapy as a one-shot approach to restore near-normal hearing. The need to deal with a large coding sequence exceeding the packaging capacity of adeno-associated virus (AAV), standard gene therapy vectors, has led to the use of dual-AAV or overloaded AAV approaches^36–38^ both of which have been demonstrated the ability to partially recover hearing in *Otof*-KO mice. SV replenishment remained below the normal speed in transduced hair cells, though, which might reflect the fact that exogenous otoferlin expression by dual AAV-mediated gene therapy did not fully achieve the normal otoferlin levels^36^.

We are currently witnessing the first trials on small numbers of children dosed with dual-AAV that provide proof of concept for clinical gene replacement therapy of the cochlea^39–41^. So far, no dose-limiting toxicity or serious adverse events have been observed. Hearing thresholds assessed by recordings of auditory brainstem responses and psychophysical pure tone audiometry improved from not measurable to near normal ~30 dB (hearing level), which is very encouraging. Yet open questions remain that would greatly benefit from further studies in mouse and NHP models of *OTOF*-related auditory synaptopathy. For example, the efficacy of clinical gene therapy could be limited, which may only be detected late as a slow and potentially incomplete speech development. Moreover, the reliability and stability of hearing restoration by *OTOF* gene therapy remain to be determined. Finally, optimizing the i) capsid-promotor combinations, ii) immunomodulation protocol and iii) regimen for treating both ears remain important tasks.

**Fig. 1:**
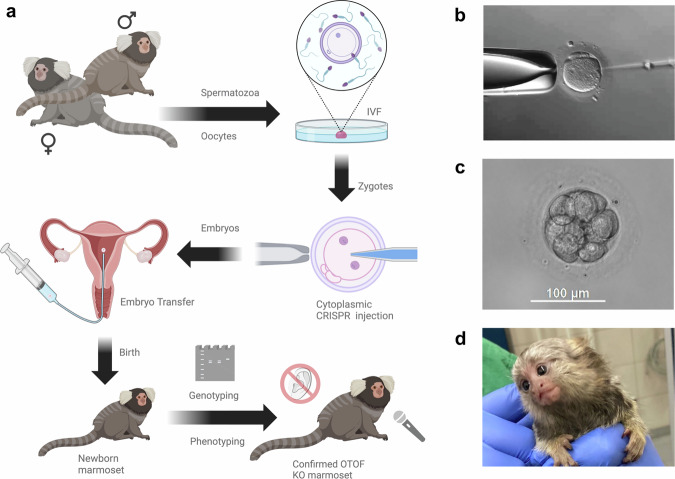
Overview of the generation and characterization of *OTOF*-knockout (KO) marmosets. **a** Workflow used to generate the *OTOF-*KO marmosets. Quantitative details (counts and percentages) are provided in Table 2. **b** Injection of the CRISPR components into a fertilized oocyte (zygote). **c** A 12-cell stage marmoset embryo. Vital embryos around the 12-cell stage were usually transferred to surrogate mothers. **d** Marmoset monkey born after embryo transfer. Workflow created in BioRender. Kahland, T. (2025) https://BioRender.com/g4zkenk.

To close this gap, we generated *OTOF*-KO marmosets combining in vitro fertilization and CRISPR/Cas9 gene editing in zygotes and demonstrated them to be non-mosaic models of *OTOF*-related auditory synaptopathy (Fig. 1).

## Results

### Validation of the marmoset *OTOF*-specific guide RNAs

**Fig. 2:**
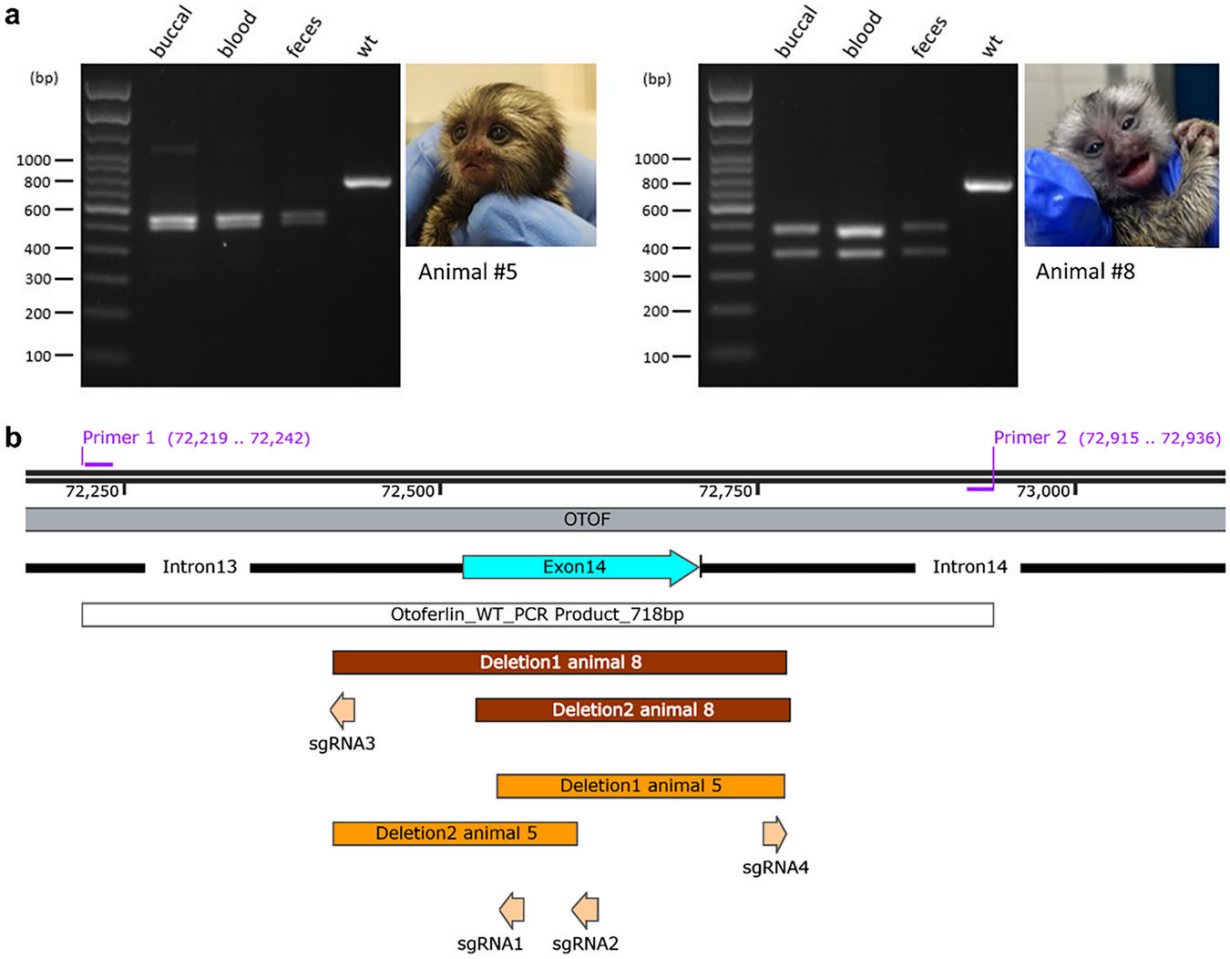
Genotyping overview of animals 5 and 8. **a** PCR genotyping of DNA samples from all three embryonic germ layers from animals #5 and #8 compared to marmoset wildtype (wt) DNA. Each PCR was performed with primers 1 and 2 flanking the region containing the sgRNA binding sites. **b** Sequence map showing the deletions affecting exon 14 of the *OTOF* gene in the two animals #5 and #8. Alignment of the sequence map was done according to the published *C. jacchus* genome (NC_071455.1 Chromosome 14, mCalJac1.2pat.X, INSDC Assembly GCF_011100555.1; *OTOF* gene ID: 100408695). A detailed analysis of the *OTOF* alleles is shown, indicating the activity of all 4 sgRNAs during the generation of the 4 different alleles detected in the two *OTOF*-modified offspring. Sequences were obtained from TOPO-cloned PCR products. PCRs for sequencing were performed with primers 3 and 4 (Supplementary Fig. 2), located outside of the displayed region.

Based on previous work in mice and NHPs^42,43^, we employed a CRISPR/Cas9 strategy with multiple single guide RNAs (sgRNAs) targeting a functionally essential part of the *OTOF* gene. Four sgRNAs (Table 1) were selected using the guide RNA selection tool CRISPOR^44,45^ to target the exon 14 of the marmoset *OTOF* gene, analogous to a successful KO strategy in mice^29,46^. The target sites of sgRNA1 and 2 are located within exon 14 and the target sites of sgRNA 3 and 4 are located upstream and downstream of exon 14, respectively (Fig. 2). The functionality of the selected four sgRNAs used in this study was validated in vitro using pre-formed Cas9/sgRNA-ribonucleoprotein (RNP) complexes and a PCR product amplified from the *OTOF* gene target region. The cleavage efficiency of all four sgRNAs was then analyzed by fragment analysis using high-resolution capillary electrophoresis (CE). To further validate the functionality of these sgRNAs, marmoset fibroblasts were transfected with Cas9/sgRNA RNP complexes of sgRNAs 1 and 2 and analyzed by target region amplification, followed by fragment analysis as described above. In addition, PCR products were also subcloned to obtain single-clone DNA sequences. Sequencing revealed deletions between the sgRNA target sites, confirming the intended *OTOF* modifications in marmoset fibroblasts. In conclusion, the suitability of sgRNAs 1, 2, 3, and 4 to delete relevant parts of the functionally essential exon 14 of the marmoset *OTOF* gene was proven before their use in embryos.

**Table 1:** sgRNAs

name	direction	location	protospacer sequence
sgRNA1	antisense	exon 14	5’-CGGGCCCACUGGCGUUCUGG-3'
sgRNA2	antisense	exon 14	5’-AUGAGGCUUGUGUUCAUACG-3'
sgRNA3	antisense	upstream exon 14	5’-AUGGUCUCUAAGGGACGGCU-3’
sgRNA4	sense	downstream exon 14	5’-AAGUGCAAGCCGAUGGGCGU-3’

### Numbers of oocytes and experimental groups

Altogether, we performed 73 oocyte collection procedures (combined ovary punctures and ovariectomies) and collected 1825 oocytes that were considered to have developmental potential. Oocytes with an insufficient diameter (<80 µm) and apparently degenerating oocytes (granulation and fragmentation) were discarded and not further considered. Oocytes that were already mature (MII oocytes) at the time of recovery were also excluded as they had, for currently unknown reasons, no developmental potential in our experimental setting^47^. The oocytes were used in three different experimental groups (Table 2). All experimental strategies primarily aimed at preventing genetic mosaicism and off-target modifications, as such effects are particularly disadvantageous in species with long generation times such as NHPs^48^. Therefore, the CRISPR/Cas9 mix was based on short-lived protein and RNA components (RNP complexes) instead of stable DNA components. In Group 1, we tested pre-IVF injection of the CRISPR/Cas9 components at a concentration of 15–20 ng/µl Cas9 protein (proven to be sufficient in mouse embryos) and each of the four sgRNA at 10 ng/µl into in vitro matured oocytes. In Group 2, we tested post-IVF injection of the CRISPR/Cas9 components at a low concentration of 15–20 ng/µl Cas9 protein and 10 ng/µl of each sgRNA. In addition, some injection mixtures in Group 2 (see section “Determination of suitable concentrations of CRISPR/Cas9”) were supplemented with 25 ng/µl mRNA of *hCas9* (human codon-optimized Cas9 nuclease). In Group 3, we used a higher concentration of 100 ng/µl Cas9 protein, 25 ng/µl *hCas9* mRNA and 15 ng/µl of each sgRNA for injection post fertilization. The injection volume cannot be accurately determined in our setup. Based on the duration of the injection and the flow-rate we estimate it to be in the range of 1–5 pl.

**Table 2:** Overview of the numbers and efficiencies of the different steps of the experimental procedure in the 3 different experimental groups: oocyte injection with low CRISPR component concentration, zygote injection with low CRISPR component concentration, and zygote injection with high CRISPR component concentration

	Oocytes low conc.(Group 1)	Zygotes low conc.(Group 2)	Zygotes high conc.(Group 3)	Total
Number of oocyte collections (combined OPUs and OvHs)	11	20	42	73
Number of collected oocytes	317	515	993	1825
Number of in vitro matured oocytes (MII) used for injections	98	166	374	638
Number of developed embryos (% cleavage rate)	11 (11.2)	54 (32.5)	196 (52.4)	261 (40.9)
Number of transferred embryos	3	22	56	81
Number of embryo transfers (ET)	2	9	20	31
Number of pregnancies; all resulted in deliveries (% pregnancy rate)	0 (0)	3 (33.3)	3 (15)	6 (19.4)
Number of newborns (% newborns per ET)	0 (0)	4 (44.4)	4 (20)	8 (25.8)
Number of KO animals (% KO per ET)	0 (0)	0 (0)	2 (10)	2 (6.5)

### Determination of suitable stages for micro-injection of the CRISPR/Cas9 components

In mice, CRISPR/Cas9 components are usually injected into zygotes. However, this can result in genetic mosaicism if the CRIPSR/Cas9 components are still active beyond the zygote stage^48–50^. To reduce the probability of mosaicism, we first aimed to inject the CRISPR/Cas9 components into the mature oocyte before IVF. We reasoned that the CRISPR/Cas9 activity remaining in the oocyte would also delete the sperm-delivered *OTOF*-allele after fertilization. We collected 317 oocytes. 98 in vitro matured MII oocytes were injected (Table 2). However, in our set-up, the oocytes injected before IVF generally lost their developmental competence and hardly developed into embryos. Out of 98 oocytes injected, only 3 oocytes developed into embryos, which were transferred to surrogate mothers. No pregnancy resulted from these three embryos. Due to this finding, this approach was not pursued further.

### Determination of suitable concentrations of CRISPR/Cas9

**Fig. 3:**
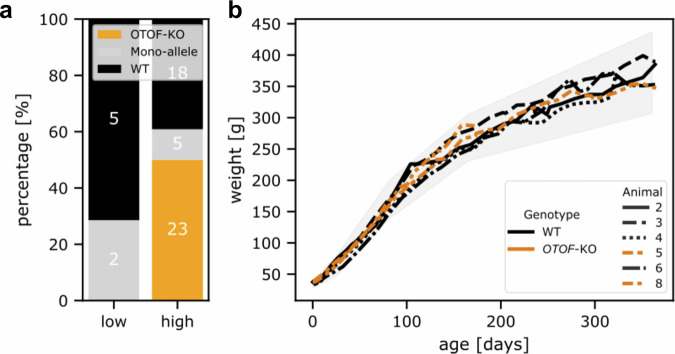
Efficiency of CRISPR/Cas9 *OTOF* editing and normal body weight gain pattern of the produced offspring. **a** With the high CRISPR concentration, the majority of the embryos showed a genetic modification of the *OTOF* locus while with the low concentration no complete *OTOF*-KO was achieved. Numbers inside the bars correspond to the absolute number of embryos analyzed. **b** Development of body weight of *OTOF*-KO (orange) and WT (black) animals within the first year of life in the present study. The gray shaded area shows the minimum and maximum weight observed in reference data from WT animals of an earlier project generating GFP-transgenic animals, which were obtained after transfer of naturally conceived embryos^89^.

In the next step, we tested post-IVF injection of the CRISPR/Cas9 components into zygotes (Group 2). In mice, usually 15–20 ng/µl Cas9 protein is sufficient to achieve DNA deletions in zygotes^51–54^. In Group 2, we collected 515 oocytes and obtained 166 putative zygotes, which were injected with a CRISPR/Cas9 mix with 15–20 ng/µl CRISPR/Cas9, and 10 ng/µl of each of the four sgRNAs. In two out of 20 experiments in Group 2, injection mixture was additionally supplemented with 25 ng/µl hCas9 mRNA, but none of the resulting three embryos was selected for transfer. The post-IVF injected embryos were developmentally competent: of the 166 injected zygotes, 22 developed into vital embryos. These embryos were transferred in nine embryo transfers (ET, Table 2) usually at the 6–12-cell stage, to surrogate mothers. Seven other embryos showed a significantly delayed developmental progress in vitro or even an arrest, were not considered vital, and were not used for ET. These delayed/arrested embryos were used for *OTOF*-genotype analysis. Only two (28.6%) of them had a mono-allelic *OTOF* modification and none were *OTOF*-KO (Fig. 3a). Transferred embryos led to three pregnancies in Group 2, resulting in three live newborns (one pair of twins and one singleton) and one stillbirth (most likely due to intracranial bleeding). Genotyping of these animals (animals #1–4) did not show any modification of the *OTOF* gene (see Table 3).

**Table 3:** List of offspring obtained, along with additional information

DOB	Animal ID in this study	Sex	Experimental Group	Genetic status	(Current) Status
21.01.2023	1	M	Group 2	*WT*	Stillbirth
16.02.2023	2	M	Group 2	*WT*	10.01.2025 Planned euthanasia for organ harvesting
04.04.2023	3	F	Group 2	*WT*	10.01.2025 Planned euthanasia for organ harvesting
04.04.2023	4	M	Group 2	*WT*	16.01.2025 Planned euthanasia for organ harvesting
03.05.2023	5	F	Group 3	*OTOF-KO*	05.05.2024 Euthanasia due to anesthesia complication
20.07.2023	6	M	Group 3	*WT*	16.01.2025 Planned euthanasia for organ harvesting
20.07.2023	7	M	Group 3	*WT*	25.07.2023 Peracute death due to septicemia
29.07.2024	8	M	Group 3	*OTOF-KO*	04.09.2025 Breeding permission obtained

*WT* wild type, *OTOF-KO* otoferlin knock-out.

Based on these findings (Fig. 3a), we increased the concentration of the CRISPR/Cas9 mix to 100 ng/µl Cas9 protein, 25 ng/µl hCas9 mRNA, and 15 ng/µl of each sgRNA. A total of 993 oocytes were collected, of which 374 were successfully matured in vitro into MII oocytes (Group 3). These 374 putative zygotes were injected resulting in 56 viable embryos that were transferred to surrogates in 20 ETs (Table 2). Optimization of the CRISPR/Cas9 mix was performed along with refinement of the injection technique, which included optimization of the microinjection flow rate, speed of needle insertion and withdrawal, depth of needle insertion. This improved the overall survival rate of zygotes and embryos, resulting in a higher embryo development rate (Table 2). The transfers resulted in three pregnancies in Group 3, and four newborns were delivered (one pair of twins and two singletons). One animal from the twin birth died 5 days after birth presumably due to immaturity-associated birth defects, such as a congenital cardiac septal defect and increased gut permeability which ultimately led to septicemia. Upon genotyping, this animal (animal #7) and animal #6 showed no genetic modification, while animals #5 and #8 showed only two bands of significantly smaller size than the PCR amplicon of the WT control. This indicates a compound heterozygous genotype of these animals showing a deletion of significant portions of *OTOF* exon 14 (Fig. 2a). Capillary electrophoresis was performed to confirm the results and to determine the lengths of the *OTOF*-deletions more precisely. The results showed deletions of 192 bp and 227 bp in animal #5 and deletions of 247 bp and 357 bp in animal #8 and no additional peaks indicating mosaicism were detected (Fig. 2b, Suppl. Figure 1). The deletions in animal #5 are located between the target sites of sgRNAs 2 and 3 and between sgRNAs 1 and 4, respectively. Animal #8 showed larger deletions compared to animal #5 on both alleles, corresponding with the target sites of sgRNAs 1 and 4 and sgRNAs 3 and 4, respectively. Results were validated by PCR products obtained from more distally located primers 3 and 4, excluding larger deletions. These PCR products were subcloned to obtain single-clone DNA sequences. The corresponding data confirmed the four different *OTOF* alleles in the two postnatal animals #5 and #8 (Suppl. Fig. 2).

We were able to sample cells and genomic DNA from all three embryonic germ layers (buccal cells— ectoderm, blood cells—mesoderm, feces containing gut epithelial cells—endoderm) and found the same pattern of PCR bands in all samples (Fig. 2a). Importantly, this indicates that the modification of the *OTOF* gene occurred already at the single cell (zygote) stage, without genetic mosaicism. Additionally, we were able to confirm the presence of the same genetic modification in the sperm sample collected from animal #8 at the age of 14 months (Suppl. Fig. 3), which allows further use of this animal for establishing the colony of OTOF-deficient marmosets.

In addition, 53 non-vital (arrested) embryos between the 8-cell and blastocyst stages were used for PCR analysis of *OTOF* gene modifications to obtain more robust data on the modification efficiency. From 46 of these embryos, we obtained enough DNA for analysis. Half of these embryos (50%) showed a putatively complete KO of the *OTOF* gene with no WT band. Five additional embryos showed at least one mutated allele (11%). In 18 of the embryos (39%) no modification was detected (Fig. 3a). In summary, 28 of the 46 arrested embryos showed an *OTOF* modification (61%).

### No off-target modifications were detected

The 16 bioinformatically most likely off-target sites were analyzed in animal #5 and no modifications were detected compared to the WT reference sequences (Suppl. material 1). These data demonstrate, at least for this animal, that the selected sgRNAs were highly efficient and specific, as bioinformatically predicted (see “Methods”).

### Phenotyping of the *OTOF*-KO marmosets

The *OTOF*-KO newborns were raised by foster parents. There were no apparent differences in WT mother–offspring pairs in the colony. Newborn *OTOF*-KO animals were breast-fed and gained weight reaching the 62th and the 69th percentile at 6 months of age and the 36th percentile at 12 months of age of comparison animals (Fig. 3b). There was no effect of genotype on body weight (one-way ANOVA of *OTOF*-KO vs. WT and comparison animals at 6 months of age: *F*(1, 13) = 0.59, *p* = 0.456).

### Auditory synaptopathy in *OTOF*-KO marmosets

**Fig. 4:**
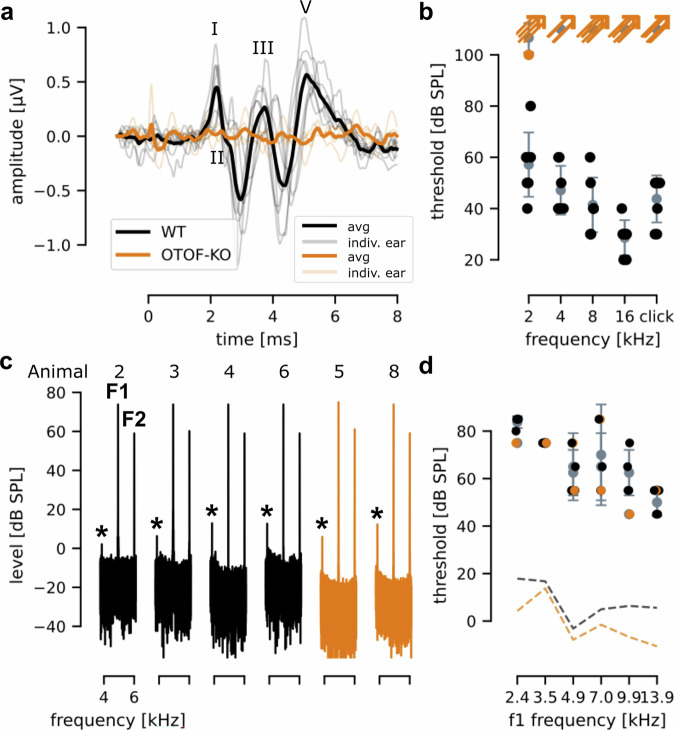
Assessing hearing of *OTOF*-KO marmosets with ABR and DPOAE recordings. **a** ABR waveforms for 80 dB SPL clicks for WT animals (black, *n* = 4 animals, 8 ears) and 100 dB SPL for *OTOF*-KO animals (orange, *n* = 2 animals, 4 ears). Individual animals are plotted in light, thin lines and averaged data are presented with thick lines for both genotypes. WT ABR waveforms displayed several identifiable peaks (indicated by roman numerals), whereas the *OTOF*-KO responses were flat. The color code (WT – black; KO – orange) is used throughout all subpanels **b** WT animals (black filled circles) displayed typical frequency-dependent thresholds with lowest thresholds of 28 ± 6.9 dB SPL (mean ± STD) observed at 16 kHz. In contrast, in all but one case (orange filled circle), no threshold could be determined for *OTOF*-KO animals (orange arrows). Mean and standard deviation are plotted in gray. **c** DPOAEs recorded in all animals (individual panels) for stimulation with primary frequencies F1 = 4.92 kHz and F2 = 6 kHz presented at a level of 75 dB SPL (F1). The cubic distortion product (asterisk) at 3.84 kHz is clearly visible for all WT (black, *n* = 4 animals, 4 ears) as well as *OTOF*-KO animals (orange, *n* = 2 animals, 2 ears). **d** DPOAE thresholds of individual ears (KO: filled orange, *n* = 2 animals, 2 ears) and WT: black circles, *n* = 4 animals and 4 ears) were plotted as a function of F1 frequency. At an F1 of 13.9 kHz thresholds were lowest at 51 ± 5.5 dB SPL (mean ± STD). Mean and standard deviation are plotted in gray. The measured noise floor is plotted with dashed lines.

Starting at 6 months of age, all live offspring were tested for hearing status. In terms of modeling *OTOF*-related auditory synaptopathy, we expected failure to activate the auditory pathway but normal function of the ear upstream of IHCs^27^. We therefore recorded both auditory brainstem responses (ABR; Fig. 4a,b) as well as distortion product otoacoustic emissions (DPOAE, Fig. 4c,d) under general anesthesia. Individual ABR waves were reliably observed in all ears from WT animals in response to clicks (Fig. 4a) and pure tones. In contrast, no waves were observed in *OTOF-*KO animals for clicks even up to a sound level of 100 dB (SPL peak equivalent) (Fig. 4a). For stimulation with pure tones, unexpectedly, an isolated ABR-like response was observed in one *OTOF-*KO ear following stimulation with 2 kHz tone bursts starting at 100 dB (SPL). This response was observed for 2 kHz tone bursts regardless of their phase, indicating that it does not reflect a microphone potential of hair cells. The response showed peak 1 and 2 latencies compatible with those of ABR waves I and II at 80 dB (SPL) in WT marmosets (Suppl. Fig. 5). In no other case, an ABR was observed (Fig. 4b). In WT animals, ABR thresholds (Fig. 4b) were frequency dependent (repeated measures ANOVA: *F*(4,24) = 6.76, *p* = 0.00086). For statistical evaluation, thresholds were set to 110 dB SPL if no response was observed (Fig. 4b, Supplementary Material 2).

DPOAEs were readily observed in *OTOF-*KO animals with good amplitudes (Fig. 4c) demonstrating normal auditory function upstream of synaptic sound encodings. DPOAE thresholds (Fig. 4D) were comparable between WT and KO animals. A repeated measures ANOVA including within-subject factor frequency and between-subject factor genotype revealed a significant main effect of frequency (*F*(5) = 8.614, *p* = 0.002) but not of genotype (*F*(1) = 1.086, *p* = 0.424). Further analysis can be found in supplementary materials (Suppl. Material 2).

### Largely unaltered vocal repertoire

**Fig. 5:**
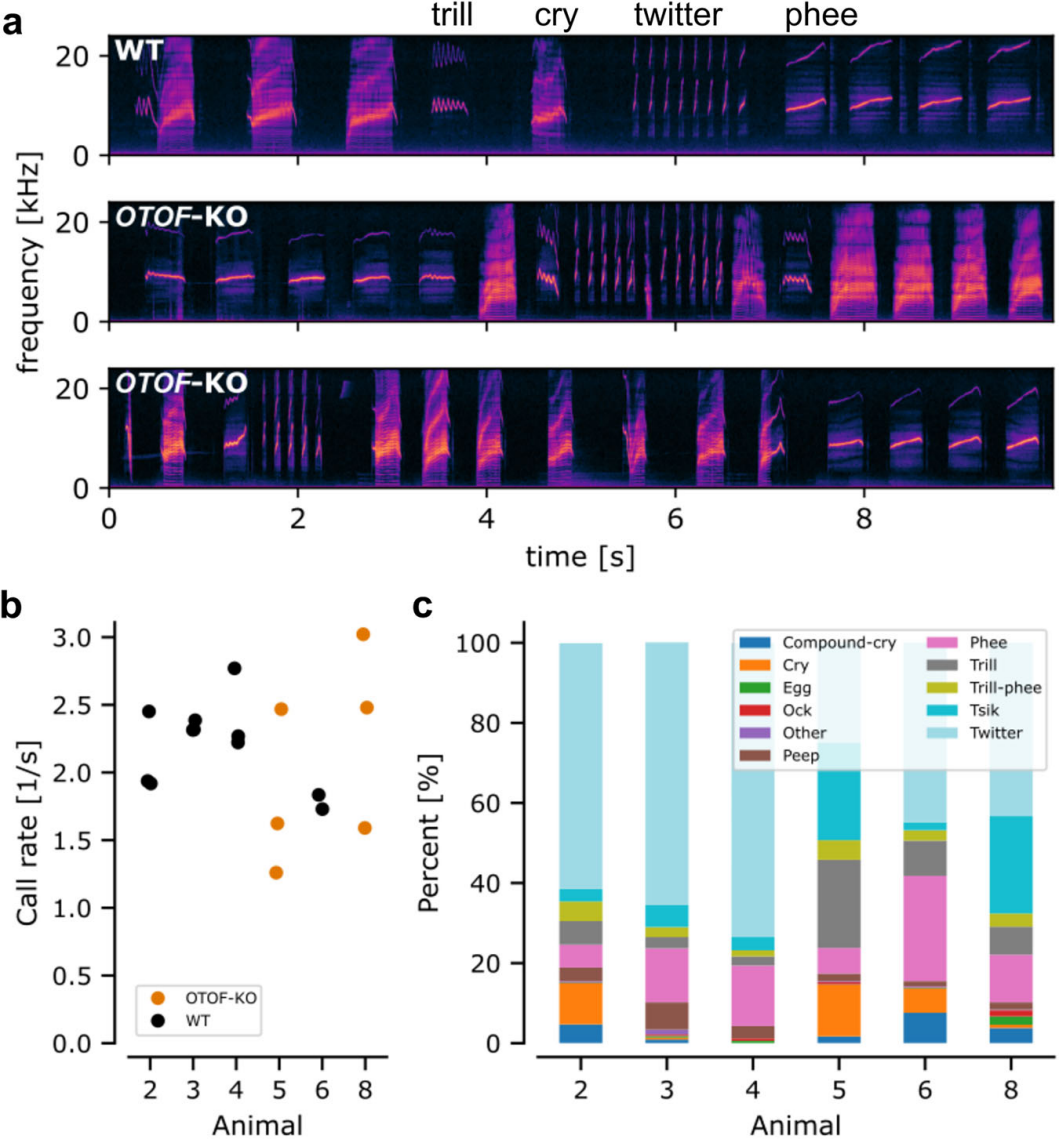
Analysis of early postnatal vocalization behavior in WT and *OTOF*-KO animals. **a** 10 s long example spectrograms of one wildtype and both *OTOF*-KO animals. Both, WT and *OTOF*-KO animals vocalized readily and displayed a diverse set of vocalizations including compound-cries, cries, trills, twitters, phees, and tsiks. **b** For each recorded session (filled circles), we determined the calling rate split between animals. The calling rate was comparable between WT (black circles) and *OTOF*-KO (orange circles) animals (ANOVA: *F*(1, 15) = 0.263, *p* = 0.615). **c** Displays the distribution of call types for each animal recorded in postnatal weeks 2 to 4. A total of 3844, 4499, 4218 and 2154 vocalizations were classified for WT animals #2, #3, #4, and #6, respectively. Analysis of *OTOF*-KO animals was based on 3231 and 4261 vocalizations for animals #5 and #8, respectively. The most common call types observed were twitters, followed by tsiks and phees or cries.

Next, we investigated the vocalization behavior of WT and *OTOF-*KO offspring at an age of 2 to 4 postnatal weeks. Animals were temporarily taken from their family cages and placed in a dedicated recording box. During the recordings, animals stayed in visual contact with their family. As described by others^55^, infant marmoset monkeys were highly vocal and displayed a wide variety of call types including the main call types: trills, twitters and phees as well as calls typically found in infancy, such as cries and compound-cries (Fig. 5a). Intriguingly, these call types were not just observed for WT animals but also for both *OTOF-*KO animals (Fig. 5a). In total, 17 recording sessions were performed. In these, 22,207 vocalizations were recorded and detected semiautomatically. On average, WT and *OTOF-*KO animals vocalized similarly frequently (WT: 2.2 ± 0.31 calls/s [mean ± STD]; KO: 2.07 ± 0.68 calls/s [mean ± STD]; Fig. 5b). A one-way ANOVA to compare the effect of genotype on calling rate revealed no statistically significant difference (*F*(1, 15) = 0.263, *p* = 0.615). To understand the vocal repertoire of each animal, all identified calls were classified by a trained observer according to classification schemes described earlier^55,56^. All animals were observed to produce all 10 call types with the exception of WT animal 2 which did not produce any cries (Fig. 5c). For all animals, twitters were produced most often (25 to 73.4 % of all vocalizations). Both *OTOF*-KO animals produced Tsik calls as the second most abundant category (24.3 % each) whereas the WT animals produced either phees (15.1, 13.5, and 26.3 %) or cries (10.2 %) as the second most observed call type (Fig. 5c). Taken together *OTOF*-KO animals were as vocally active as age-matched wild-type controls and produced the full vocal repertoire of infant marmosets. This is reminiscent of an analysis of deaf *Otof* KO mice^57^ and other deaf mouse mutants^58^.

**Fig. 6:**
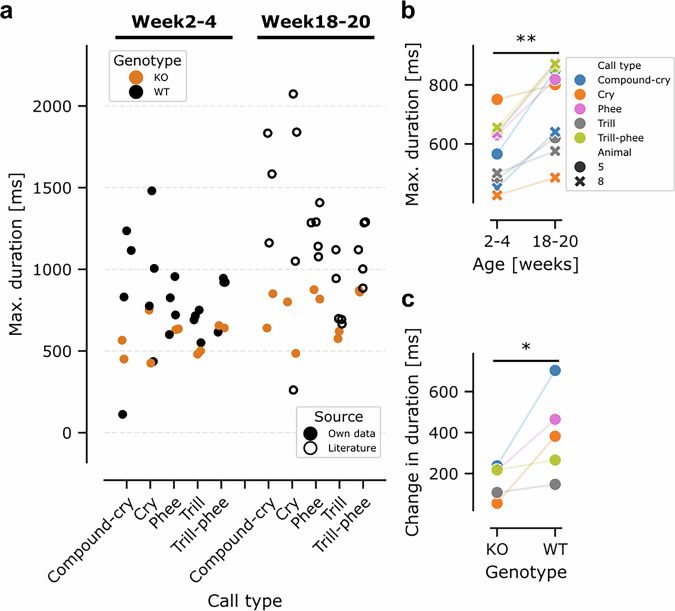
Analysis of call durations in WT and OTOF-KO animals. **a** The maximum duration of a call elicited by each WT (black) and *OTOF*-KO (orange) in week 2 to 4 postnatal as well as week 18 to 20 split between the five longest call categories (e.g. ref. ^55^). Each data point corresponds to a single animal. Own data gathered for the current study is plotted in filled circles and literature values are shown in open circles. **b** the maximum duration of calls increased with age for both KO animals (one-sided paired t test: animal 5: *p* = 0.00552; animal 8: 0.006802, *n* = 5 call types). The color scheme for different call types is the same as in Fig. 5. KO animals 5 and 8 are indicated with circles and crosses, respectively. **c** while WT and KO animals display increased maximum call duration between week 2–4 and week 18–20 postnatal, the increase observed for KO animals was significantly shorter (one-sided paired t test: *p* = 0.02546, *n* = 5 call types).

Auditory feedback has been shown to be relevant for marmoset vocal behavior in adulthood and real-time altered feedback leads to compensatory changes within a few hundred milliseconds^59^. We therefore hypothesized that if missing auditory feedback plays a role during infancy it should mostly affect longer duration calls. Consequently, we analyzed call durations of the five longest call types^55^: compound-cry, cry, phee, trill and trill-phee (Fig. 6a). Typically, the maximum call durations of KO animals were shorter than for WT animals. Further vocalization recordings for OTOF-KO animals in the postnatal weeks 18 to 20 revealed a similar picture when compared to data from the literature^55^. Note that for WT animals, no further vocalization recordings were performed after genotyping in the current work. While the maximum duration of vocalizations increased for all five call types (paired one-sided t-test animal #5: *t*(4) = −4.474, *p* = 0.0055; animal #8: *t*(4) = −4.208, *p* = 0.0068; Fig. 6b), they did not increase to the same extent as in WT animals. We calculated the average maximum call duration for our WT animals in weeks 2 to 4 and the data from Gultekin et al.^55^ in week 18–20. Similarly, we averaged the data for our KO animals. For all five call types analyzed, the increase in maximum call duration was smaller for KO than WT animals (Fig. 6c; paired one-sided t-test: *t*(4) = −2.759, *p* = 0.025).

### Lack of otoferlin expression in the *OTOF*-KO cochlea

**Fig. 7:**
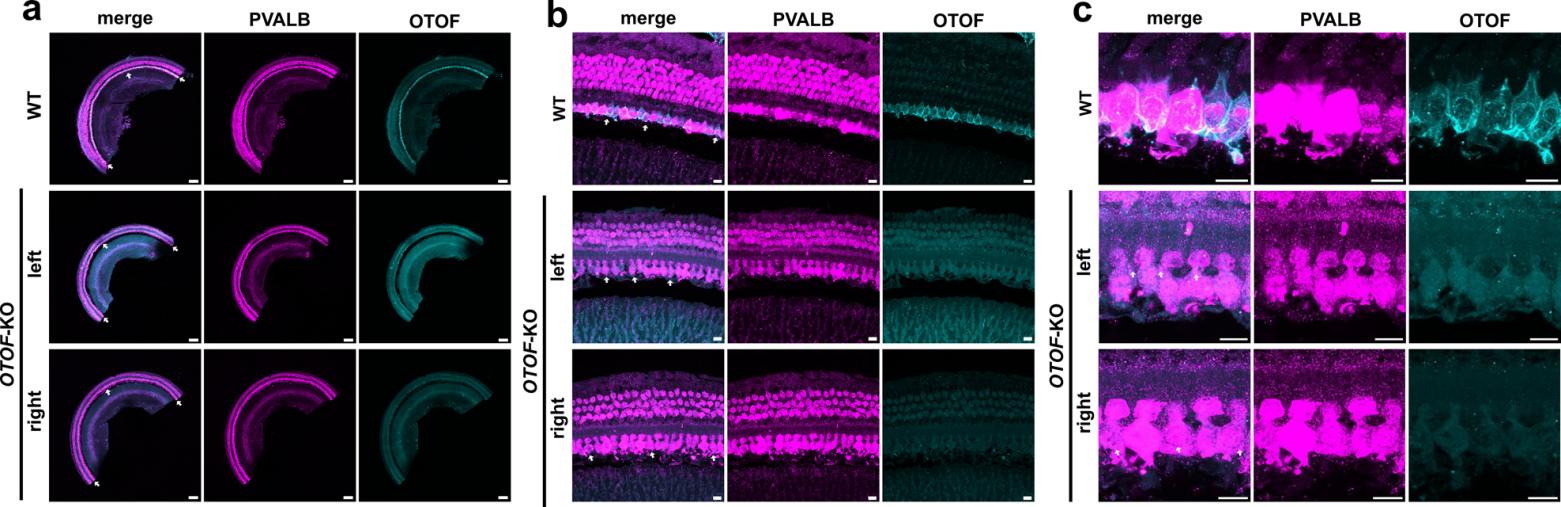
Immunofluorescence analysis of otoferlin expression in the cochlea. **a** Whole-mount of a microdissected organ of Corti from a control animal (WT) and both ears of an *OTOF*-KO animal. A piece of the midcochlear turn was immunolabeled for the hair cell marker parvalbumin (pink) as well as otoferlin (cyan). Arrows point towards the single row of IHCs that show otoferlin immunofluorescence in WT but not in the *OTOF*-KO cochleae. Scale bar: 200 µm. **b** Organ of Corti with one row of IHCs and three rows of outer hair cells shown at higher magnification. Scale bar: 20 µm. **c** close up of IHCs. Scale bar 10 µm.

We performed a postmortem analysis of otoferlin expression in the IHCs of both ears in *OTOF-*KO animal #5, which had been euthanized due to an anesthesia complication. While otoferlin expression was detected by immunofluorescence in IHCs of a control cochlea, no otoferlin expression was observed in IHCs of the *OTOF*-KO animal (Fig. 7a, b). No difference in IHC density was observed between organs of Corti derived from *OTOF-*KO and control animals (*OTOF-*KO: left = 10.4 IHC/100 µm and right = 10.0 IHC/100 µm vs. control ears: 10.5 ± 0.4 IHC/100 µm; mean ± std; *n* = 3 animals, 6 ears).

## Discussion

Here, we combined CRISPR/Cas9-mediated gene knockout with a reproductive medicine approach to generate an NHP model of human deafness. Genotyping and auditory phenotyping demonstrated the successful deletion of functional *OTOF* gene expression and disrupted synaptic sound encoding. We optimized the genetic modification approach and achieved an efficiency that renders future studies targeting *OTOF* and other genes in marmosets feasible. Importantly, we did not find evidence for mosaicism that had been a severe problem in previous genetic modification approaches in macaques and marmosets^43,60–63^. The auditory physiology phenocopies human *OTOF*-related auditory synaptopathy with a lack of auditory brainstem responses despite normal cochlear morphology and function of the ear upstream of IHC synapses. The deafness of the mutants did not obviously alter their vocalization behavior nor the parental care behavior.

### Generating *OTOF*-KO marmosets

Previous work had targeted *MYO7A* in rhesus macaques and yielded preliminary results indicating mosaicism^23^. The current study successfully combined efficient in vitro fertilization in marmosets^47^ with optimized CRISPR editing to yield genetically modified animals without genetic mosaicism. This overcomes a major problem of previous studies, the high rate (up to 100%) of mosaicism in marmoset embryos^60^. Gene editing with CRISPR is mostly performed by injection into the pronucleus allowing for precise gene modification in mice^64^. In contrast, in marmosets CRISPR/Cas9 reagents are typically injected into the cytoplasm^60^. Injections with Cas9-protein vs. Cas9-mRNA resulted in a higher likelihood of target gene modification by nuclease treatment but resulted in a high rate of mosaicism in both cases^60^. In the current study, we optimized the concentration of the mix of CRISPR components resulting in a low rate of mosaicism, which is of great value in NHP disease models. The use of several sgRNAs flanking a region of interest is more efficient than single guides^42^, as has also been shown in NHPs^43^. CRISPR was apparently active only in the zygote as samples from all three embryonic germ layers show all the same pattern of two bands in the genotyping PCR. This indicates that active Cas9-degradation^65^ is not required for avoiding mosaicism. However, it seems to be important to use short-lived RNP complexes for the genetic modification of the zygote. Alternatives to the presented approach include cloning of NHPs from in vitro genetically modified NHP cells. However, this might yield side effects, and chimera formation is not really established in NHPs and animal consuming (embryo donors). In addition, this would still require normal NHP generation times for efficient generation of multiple offspring. While marmosets have shorter generation times than macaques^20^, it is still substantially longer than in mice and pigs. In vitro maturation of neonatal testis or ovarian tissue could potentially shorten the overall generation time. However, proper epigenetic imprinting of such germline cells and gametes is questionable. For example, spermatozoa in older marmosets display different DNA methylation patterns than spermatozoa from young adults^66,67^. The production of genetically modified NHPs will therefore remain slow and costly for the time being (see limitations section).

### Modeling human *OTOF*-related auditory synaptopathy in non-human primates

As expected for the *OTOF*-related auditory synaptopathy^26–28^, ABRs were absent even for the loudest stimuli employed (clicks of 100 dB (peak equivalent)) but DPOAEs were readily detected with good amplitudes. This indicates normal function of the ear upstream of IHC, but a complete block of sound-evoked synaptic transmission by IHCs. We currently do not know the precise mechanism underlying the isolated ABR-like response in one *OTOF-*KO ear following stimulation with 2 kHz tone bursts at levels ≥100 dB (SPL). It does not seem to reflect a microphone potential of hair cells. We currently cannot safely distinguish the possibility of residual sound encoding in the absence of otoferlin from a somatosensory or vestibular systems nature of the response. Specifically, vestibular nerve compound action potentials have been reported for *Otof* KO mice with short latencies^68^. The second *OTOF*-KO that showed the ABR-like response in one ear is required for breeding and remains to be treated with *OTOF* gene therapy, which currently precludes histological analysis.

Importantly, the parental care and overall postnatal development were not noticeably altered. While more thorough analysis is warranted, observation of the *OTOF*-KO animals indicated overall normal social behavior. Despite deafness according to ABR, loudness, frequency, types, and prevalence of vocalizations seemed largely unchanged early postnatally (Fig. 4b; Fig. 5b, c). This supports the notion that these vocalizations are a largely innate behavior and, at this level of analysis, do not require hearing experience and auditory feedback. We note that the hearing assessment was limited to physiological estimates which, however, do not safely exclude a limited amount of residual hearing. Hence, while perception of vocalizations is very unlikely, future psychophysical assessment will need to assess residual hearing.

Due to their loquacious nature, marmosets have recently become a focus of studies on the evolutionary origin of human speech and language. Large changes in vocalizations have been documented during development where many of these are maturation driven^16,17^. Evidence of vocal learning is scarce and generally limited to the learning of the use of the vocal repertoire or vocal accommodation^17,69^. Here, the role of hearing itself has been studied either deafening infants early on^70^, by raising infants in acoustic isolation without parents^71^ or in a single case in a squirrel monkey with early onset of deafness of unknown etiology^72^. As hearing onset is embryonal in primates and attenuation of sounds in the womb is less pronounced than thought^73^, this leaves the possibility that vocalizations in the auditory environment of the embryo are heard and lead to changes, i.e. drive the infant to produce these heard vocalizations. In this regard it is noteworthy that marmosets fetuses practice vocalizing already in the womb similar to humans^74^. However, auditory feedback has been shown to be required for precise and correct vocal pattern generation^59,75^. Interestingly, we observed subtle differences between KO and WT animals regarding the maximum call duration across various call types (Fig. 6). Here, *OTOF*-KO animals vocalized shorter than WT control animals. While KO animals increased their call durations over time (Fig. 6b) as expected during maturation^76^, the increase in duration remained below what would be predicted from WT animals (Fig. 6c). This suggests a role for auditory feedback besides maturation and might point to different developmental trajectories in deaf vs. normal hearing marmosets. Future work will need to address the fine-scale changes of vocalizations in deaf vs. normal-hearing marmosets in detail.

### Value of *OTOF*-KO marmosets for future work and limitations of the present study

Mutations in the *OTOF* gene and its resulting deafness DFNB9, account for 1–8% of hereditary deafness cases^77^. This substantial patient population has now come into focus of clinical trials^39,40,78^. Results from these trials report restoration of auditory function via auditory brainstem responses but critically also restoration/establishment of auditory perception and some form of speech understanding^39,40^. While these are tremendous achievements, unsuccessful, i.e. non-functional gene therapy cases were also observed in the same trials^39^. Potential explanations can be found in earlier rodent preclinical trials which allow to link functional restoration to hair cell physiological parameters. Both, overload as well as dual AAV approaches restored ABR responses but failed to restore ABR amplitudes to WT levels^36–38,79^. This could be explained by the correlation of hair cell exocytosis and otoferlin expression levels: WT levels of exocytosis are achieved with expression levels of 70 % of the WT otoferlin expression^51^. Overall, this indicates that graded restored expression of *OTOF* also leads to graded functional restoration.

In this context, *OTOF*-KO NHP models could play a critical role in improving *OTOF*-related gene therapy before moving to clinical trials in humans, complementing the highly relevant work in mouse mutants. NHP models will allow for extended studies on the specificity, efficacy and longevity of the functional restoration upon gene therapy. For example, otoferlin levels in IHCs and target-cell specificity of transgenic otoferlin expression can now be assessed for different capsid-promotor combinations by post-mortem histology of the treated *OTOF-*KO marmoset cochlea. This will allow to correlate auditory function following *OTOF*-related gene therapy assessed at the physiological and behavioral level with postmortem analysis of otoferlin expression in IHCs, which in turn will allow to optimize dosing, vectors, promotors, transgene and AAV-administration. Moreover, NHP models will support late-preclinical studies on the dependence of the functional outcome on intervention timing, AAV-dose, presence of neutralizing antibodies, and immunomodulation. In addition, *OTOF*-KO marmosets could be useful for the development of next-generation cochlear implants^80,81^. Further, this model uniquely enables exploration of how auditory feedback shapes vocal development, thereby deepening our understanding of basic neuroscience and driving translational clinical research into auditory disorders. We conclude that the key role of appropriate NHP models of genetic deafness for late-preclinical testing of novel therapies offset the challenges such as slow generation time, risk of off-target effects of CRISPR/Cas9, potential immune responses to Cas9, high costs and ethical concerns regarding disease modeling in NHPs.

Only two *OTOF*-KO animals were obtained from 81 embryos transferred in the present study. We achieved a major gain in efficiency from learnings such as the success with increased CRISPR activity. Our most efficient protocol resulted in rates of 20% genetically modified animals per transfer and of ~7% per transferred embryo (Supplementary Table 1), which is comparable to or exceeds most published values^82^. Future work will obtain further animals using the optimized protocol and from cross-breeding existing *OTOF-*KOs with wildtype marmosets. Parallel efforts of generating *OTOF*-mutant macaques from a spontaneously arisen mutation will help serving the urgent needs described above^83^. This notwithstanding, the generation times will remain limiting for the availability of the NHP models of DFNB9. Further challenges of our approach concern the risk of off-target CRISPR effects. We consider it low due to the short-lifetime of hCAS9 protein and mRNA as compared to plasmid-coded hCas9. Moreover, the Cutting Frequency Determination (CFD) specificity score was above 90 out of 100 for all four guide RNAs indicating a very low probability for off-target effects^84,85^ and the 16 bioinformatically most likely off-target sites were found to be unaltered (Suppl. Material 1). Overall, we consider the risks and disadvantages associated with the production and use of genetically modified NHPs to be largely controllable and acceptable in relation to the expected benefits of these animals for biomedical research and translational therapy development.

## Methods

Detailed protocols for animal housing, reproductive cycle monitoring, oocyte retrieval and handling, sperm retrieval and in vitro fertilization, as well as CRISPR/Cas9 related methods are also described in our earlier work^47^ and are summarized here briefly. Unless otherwise mentioned, media components were purchased from Sigma-Aldrich (Merck), Darmstadt, Germany.

### Animals, housing conditions, and ethics

All animals were housed and cared for in accordance with institutional guidelines and the German Animal Welfare Act. Adult *Callithrix jacchus* monkeys from the German Primate Center were used as gamete donors. Daily health monitoring and veterinary assessments ensured animal welfare. The housing environment included climate-controlled rooms, a 12-h light/dark cycle, and enrichment through structured cages and diverse dietary supplementation. Experimental approval was granted by the Lower Saxony authorities (LAVES License #33.19–42,502-04–19/3221).

### Female reproductive cycle monitoring

Ovarian cycles were assessed via biweekly plasma progesterone measurements using a validated enzyme immunoassay. Luteolysis was induced with 1.5 µg PGF-2α (Estrumate, Essex Tierarznei, Munich, Germany) per animal to synchronize cycles. Hormonal stimulation began the next day with daily administration of 25–35 IU recombinant human FSH (Gonal-f, Merck Europe B.V., Amsterdam, the Netherlands) for 6–9 days (dose and duration of stimulation were individually adjusted) with or without subsequent administration of 75 IU hCG (Ovogest, MSD Animal Health, Unterschleissheim, Germany)^47^^.^

### Oocyte retrieval procedures

All marmoset oocyte donors underwent three ovum-pick-up (OPU) cycles followed by an ovaryhysterectomy (OvH). Anesthesia protocols involved either ketamine/medetomidine/atropine or alfaxalone/midazolam/buprenorphine combinations. Oocytes were aspirated using a Labotect system or dissected from excised ovaries post-OvH. Pain management and antibiotics were administered perioperatively.

### Culture conditions and plate preparation

Culture plates were prepared the day prior to use, with drops of stage-specific medium under light mineral oil (LiteOil®, LifeGlobal Europe, Brussels, Belgium) at the stage-specific gas environment. Plates were incubated to stabilize pH and oxygen conditions before use.

### Oocyte handling and in vitro maturation

Collected immature oocytes were cultured in POM medium (CosmoBio, Tokyo, Japan) supplemented with 5% FBS (Biochrom AG, Berlin, Germany), 1 IU/mL FSH and 1 IU/mL hCG under light mineral oil for 29–30 h at 37.5 °C, 5% CO_2_ in air.

### Sperm collection and in vitro fertilization

Sperm samples were obtained using penile vibrostimulation device (FertiCare personal vibrator (Multisept ApS, Rungsted, Denmark). Ejaculates were diluted in sperm collection medium (HEPES-buffered Tyrode’s lactate with 0.25 mM sodium pyruvate and 0.3% wt/vol bovine serum albumin (BSA), pH 7.3, 37 °C) and processed by density gradient centrifugation (40/80 PureSperm, Nidacon, Mölndal, Sweden) with subsequent capacitation. Sperm collected from animal #8 was not used for IVF but was subjected to DNA extraction and PCR analysis to confirm the presence of genetic modification. IVF was performed by combining capacitated sperm with matured oocytes in a drop of IVF medium (Tyrode’s lactate medium with 0.5 mM sodium pyruvate, 0.5 mM GlutaMAX (GlutaMAXTM, Gibco® (Merck), Darmstadt, Germany), 2 mM CaCl_2_.2H2O, 1% v/v minimum nonessential amino acid solution (NEAA 100x, PAN Biotech, Aidenbach, Germany), 0.3% wt/vol BSA, 10 IU/mL penicillin, and 10 µg/mL streptomycin (Penicillin-Streptomycin, PAN Biotech, Aidenbach, Germany) under oil.

### CRISPR/Cas9 mix composition and injection and embryo culture

Fifteen to sixteen hours after IVF, cumulus cells were mechanically removed and zygotes were assessed for the presence of pronuclei and polar bodies. A CRISPR/Cas9 mixture targeting the *OTOF* gene was injected intracytoplasmically at the pronuclear stage. Component concentrations varied slightly across experimental groups (e.g., 10–15 ng/μl sgRNAs; 15–100 ng/μl Cas9 protein; 0 or 25 ng/μl hCas9 mRNA; in both cases buffered in 5 mM TrisHCl pH7.4, 100 mM NaCl; details see section *Numbers of oocytes and experimental groups* and Tab.2), and injection timing was adapted in a subset of oocytes, which received CRISPR/Cas9 post-IVM but prior to fertilization.

Injections were performed using 1.6 μm pipettes and a FemtoJet 4i microinjector. Embryos were cultured in ORIGIO® Sequential media at 37.5 °C, 5% O_2_/ 5% CO_2_. From the 4-cell stage onward, media were changed every 2 days. From the morula stage, media was supplemented with 2.5% FBS. Embryo development was monitored daily by microscopy with image documentation.

### Embryo transfer to surrogate mothers and pregnancy monitoring

Five-day-old embryos were transvaginally transferred with 1-3 µl of ORIGIO® Sequential BlastTM medium into the time/stage-matched surrogate mother’s uterus via ultrasound-guided embryo transfer catheter (Oviraptor-kathether for embryo transfer, 20 G, Altair Corporation, Shinyokohama, Kohoku-ku, Yokohama 222-0033, Japan). Anesthesia was induced with 0.05 ml of midazolam [5 mg/ml] and alfaxalone [10 mg/ml] ad 1 ml/kg body weight, administered intramuscularly. Following embryo transfer, Regumate® (altrenogest, MSD Animal Health, Unterschleissheim, Germany) was administered for 10 consecutive days at a dosage of 0.44 mg/kg body weight, orally. From ED ~ 25 onwards ultrasound imaging of implantation was performed using LOGIC™E (GE HealthCare, Illinois, Chicago, USA) to confirm or exclude pregnancies. Subsequent monitoring at weekly intervals showed consistent physiological growth and development of the embryos/fetuses. After natural birth or cesarian section the surrogate parents accepted the offspring in all cases and provided care to the newborn.

### OTOF gene targeting strategy and gRNA validation

To functionally knock out the *OTOF* gene with a very high probability on both alleles, four sgRNAs were designed to target the exon 14 of the marmoset *OTOF* gene. The target sites of sgRNA 1 and 2 are located within exon 14 and the target sites of sgRNA 3 and 4 are located upstream and downstream of exon 14, respectively (see Fig. 2b). The functionality of these sgRNAs was validated in vitro using pre-formed Cas9/sgRNA-ribonucleoprotein (RNP) complexes and a PCR product amplified from the *OTOF* gene target region. The cleavage efficiency of all four sgRNAs was then analyzed by fragment analysis using gel electrophoresis and high-resolution capillary electrophoresis (CE) on a 3730xl DNA Analyzer (Applied Biosystems, now Thermo Scientific). To further validate the suitability of these sgRNAs, marmoset fibroblasts were transfected with RNP complexes of sgRNAs 1 and 2 and analyzed by target region amplification, followed by fragment analysis as described above. In addition, the PCR products were subcloned to obtain single-clone DNA sequences. Sequencing revealed deletions at the sgRNA sites, confirming the targeted gene modifications by CRISPR/Cas9 in marmoset fibroblasts.

### DNA sampling and Genotyping

Genomic DNA (gDNA) was extracted from blood (mesoderm), buccal mucosa (ectoderm) and intestinal epithelial cells (endoderm) recovered from fresh marmoset feces. In one neonate, also a piece of the umbilical cord could be obtained for genotyping. The PCR was performed using KOD Xtreme Hot Start DNA polymerase (Merck, Darmstadt, Germany) with primers (#1: 5’-CCCAGTGTACAATCTATAAACCAC-3’, #2: 5’-ATTTGTCTCCTCCACTCTTCCC-3’) flanking the sgRNA target sites. PCR was performed for 40 cycles of denaturation at 94 °C for 10 s, annealing at 60 °C for 30 s and 50 s of elongation at 68 °C. The resulting PCR products were visualized on a 1% agarose gel using GelRed® Nucleic Acid Gel Stain (Biotium, Fremont, USA). Additionally, the PCR samples were analyzed by CE to obtain the exact fragment lengths of the PCR products. Finally, the exact sequences of the mutated alleles of the genetically modified offspring were determined by Sanger sequencing. Therefore, PCR fragments derived by primers located in exon 13 and 15 (primer #3: 5’-CATAAGGCCAATGAGACCGACGAA −3’ and #4: 5’-CACGTCGTTGACCTTGTCCGAG −3’) were subcloned by TOPO cloning before sequencing. Arrested embryos were analyzed accordingly.

### Off-target analysis

Note that all gene-specific reagents (i.e., sgRNAs and hCAS9 mRNA) were used as RNA molecules and were not plasmid-coded. In this way, we intended to reduce the likelihood for off-target effects and mosaicism due to the short livetime of RNA-based reagents^84,85^ in contrast to plasmid-coded reagents. Accordingly, the CFD specificity score was 94 out of 100 (sgRNA1), 94 out of 100 (sgRNA2), 93 out of 100 (sgRNA3) and 98 out of 100 (sgRNA4), indicating a very low probability for off-target effects^84,85^. The 16 bioinformatically most likely off-target sites were analyzed (Suppl. Mat. 1). All sequence data obtained from the offspring was compared with the wild-type reference DNA sequences. Each fragment was amplified and analyzed in duplicate.

### Functional hearing tests

The functional assessment of hearing status was performed under anesthesia induced with a combination of Alfaxan (10 mg/kg BW, i.m.) and Midazolam (0.125 mg per animal, i.m.) in a dedicated surgery room for non-human primates. ABR and DPOAE recordings were done under a single anesthesia and followed procedures as described earlier^86^. For pain management, Metacam (0.2 mg/kg BW) was administered orally at least 30 min before any potentially painful procedure. Anesthesia was maintained by re-injecting Alfaxan (5 mg/kg BW) if necessary.

Monitoring of vital signs included respiratory frequency and EtCO2 with a flexible catheter placed inside a nostril (Nonin LifeSense LS1-9R). In addition, pulse oximetry was employed (Nonin LifeSense LS1-9R and Mindray iPM 12 Vet) to monitor heart rate and blood oxygenation.

Animals were transferred onto a stereotaxic frame equipped with custom ear probes mounted on ear bars. Probes allowed for acoustic stimulation with one or two loudspeakers (Etymotic ER-4PT) as well as recording via a microphone (Sennheiser MKE 2 P-C). Ear probes were gently placed into the cartilaginous part of the ear canal, taking care not to occlude the ear probe with skin. This procedure allows moving the head sideways by about 1–2 mm. Correct placement of ear probes was monitored by observing the returned acoustic stimulation on an oscilloscope (Tektronix TBS1064).

Temperature management involved two separate approaches. First, animals were placed on a warm saline bag propped on a heating pad which was kept at a temperature of 38 °C. Additionally, a forced-air blanket (pediatric plus; Bair-Hugger) was wrapped around the animal’s body. Throughout the procedure body temperature was kept between 37 and 38.5 °C by adjusting the temperature of the forced-air blanket.

For both, ABR and DPOAE recordings, calibration of acoustic stimuli was performed with 3D printed ear molds of adult common marmosets. Here, at the position of the tympanic membrane a probe microphone (G.R.A.S. 46 BE) driven by dedicated power supply (G.R.A.S 12AL) allowed to record presented sounds via a National Instruments card.

Analysis of auditory brainstem responses and distortion products was performed in Python. Statistical evaluation of ABR and DPOAE data was performed with Python and R.

### Auditory brainstem response recordings

Clicks (200 µs duration) and pure tones of various frequencies were used to evaluate auditory brainstem responses (ABRs). All acoustic stimuli were first presented at a level of 80 dB SPL. If no response was observed, the sound level of presentation was increased to a maximum of 110 dB SPL. ABRs were recorded by placing needle electrodes behind the pinna, on the vertex, and on the neck of the anesthetized animals. The difference in potential between the vertex and mastoid subdermal needles was amplified using a custom-designed amplifier, sampled at rate of 50 kHz for 10 ms, filtered (300–3000 Hz) and averaged across at least 750 stimulus presentations. The ABRs threshold was defined and determined as the lowest sound intensity for which one of the five waves was reliably visible. In case no threshold could be identified, it was set to 110 dB SPL for display and statistical evaluation.

### DPOAE recordings

Two separate headphones (Etymotic ER-3A) were used to present pure tones of two primary frequencies and sound levels. The frequency of the second primary frequency F2 was varied between F2: 3, 6, 8.5, 12, and 17 kHz. In each case, the frequency relationship between the two primary frequencies, F1 and F2 was fixed at 1.2 following earlier work by Valero et al.^87^. In addition, a level difference of 10 dB was used between F1 and F2 with F1 being louder. First, sound levels of F1 were varied in steps of 10 dB starting with 75 dB SPL and decreased to a minimum of 25 dB SPL. If no distortion product was observed at 75 dB SPL, a sound level of 85 dB SPL was presented in addition. Each frequency and level combination was presented for 4 s and the resulting microphone signal was routed through an audio interface (ZOOM UAC-2) and subsequently connected to an analog input of a National Instruments card for digitization at a rate of 200 kHz at 16 bits resolution. Putative cubic distortion products were evaluated at a frequency of ${f}_{{DP}}=2*f1-f2$. Towards this an FFT was performed on the full 4 s-long microphone signal windowed by a Hanning function. The noise floor around the distortion product was evaluated in a frequency range of ±15 Hz, excluding the putative cubic distortion product peak. DPOAE thresholds were determined as the lowest sound level of F1 at which the peak power of the putative distortion product was at least 3 dB above the noise floor.

### Vocalization recordings

Overall, procedures followed earlier work by Gultekin et al.^55^. At an age of 2–4 weeks postnatal, 2–4 recording sessions were performed with each animal. Animals were either gently taken from their caregivers inside their family cage or taken with their caregiver to a procedure room for general assessment of well-being including weighing. Afterwards, animals were transferred to a vocalization box with inner dimensions (*W *× *D *× *H*) of 22 × 24 × 30 cm. The vocalization box was located at a distance of 20 cm from the animals’ family cage. All side, floor and roof panels were made of black plastic and lined with acoustic foam (top: 7 cm, sides: 3 cm; back and bottom: 5 cm) while visual contact was possible via a transparent front panel. In one side panel, a microphone (Sennheiser MKH8020) was inserted to record the animals vocalizations via a sound card (Terratec DMX 6Fire USB) to a computer located in a separate room. Vocalizations were recorded with Audacity and stored at 96 kHz and 32 bit. Inside the vocalization box, the animal was placed on a fur—taken from the family cage—covering a hot-water bottle to assist in thermoregulation. Throughout the vocalization recordings, the animals’ and families’ behavior was monitored with two video cameras facing the transparent front panel of the vocalization box or the family cage, respectively. In general, vocalization sessions lasted 10 min and animals were returned to their family immediately afterwards. In total, the animals were separated from their family less than or equal to 12 min. Additional recordings were performed with KO animals at an age of 18–20 weeks postnatal. In this case, vocalization sessions lasted 15 min and no hot-water bottle was used. All other procedures remained the same.

Putative vocalizations were identified offline as events exceeding a certain energy threshold with custom scripts written in Matlab 2012R. Occasionally, manual adjustments to call on- or offsets as well as putative calls were made manually with Audacity. Next, putative vocalizations were classified by trained observers and followed the classification schemes presented in refs. ^55,88^ and included ten different call types: Tsik, Twitter, Phee, Trill-phee, Trill, Compound-cry, Cry, Egg, Ock and Peep. Unclear vocalizations were classified as ‘Other’. Inter-rater reliability was assessed separately for postnatal week 2–4 and week 18–20. In week 2 to 4 inter-rater reliability was based on a subset of about 12% of vocalizations (2737). 92 % of those vocalizations were classified as the same call category, leading to a Cohen’s kappa of 0.86 and an almost perfect agreement. Based on 5 % of vocalizations (1140) labeled twice by the same experimenter, the inter-rater reliability was assessed with 94 % of aligned call categories and a Cohen’s kappa of 0.91. For the later period (week 18–20), we compared 1695 vocalizations out of a total of 6122 (animal 5: 3441; animal 8: 2681). 90 % of calls were categorized into the same category, thus an almost perfect agreement between raters and a Cohen’s kappa of 0.88. Within the main text, all data presented are based on the classification by a single experimenter.

Statistical analysis of vocalization data was performed with Python and R.

### Organ of corti extraction

The cochlea was cut out of the 4% FA-fixed head using bone scissors. Tissue and bone were trimmed off with bone scissors and a rangeur till the cochlea and the labyrinth organ were visible. The cochlea was incubated in EDTA and the bone was trimmed off every two days for at least two weeks. EDTA was changed every four days. The stria vascularis, the Reissner’s membrane and the tectorial membrane were taken off with fine scissors and forceps. Afterwards, the organ of Corti was cut off the modiolus and stored in 1x PBS with 0.5% sodium azide.

### Immunofluorescence

The organ of corti was incubated in citric buffer for 5 min at 100 °C and cooled down for another 10 min. Afterwards the organ of corti was transferred to a 24 well plate covered with parafilm, so that small wells were created, and washed three times for 10 min with 1x PBS. Permeabilization was performed with 0.5% TritonX in PBS for 1 h. Unspecific binding sites were blocked with 10% goat serum, 0,5% TritonX in PBS for 1 h. The primary antibodies (rabbit anti Otof, custom SySy, chicken anti Parvalbumin, 195 006 SySy) were diluted in the blocking solution and incubated with the organ of corti overnight. The next day the samples were washed three times for 10 min with PBS. The secondary antibodies (goat anti-rabbit 633, A 21 070, Thermo Fisher; goat anti chicken 568, ab175711, abcam) were also diluted in blocking solution and incubated overnight. The organs of corti were then washed 3 times in PBS and embedded on microscope slides using spacers, round cover slips and 20 µl moviol. The slides were dried at room temperature and afterwards stored at 4 °C until imaging. The slides were imaged on a Leica SP8 confocal microscope.

### Inclusion and ethics in global research

This work was conducted entirely on the Göttingen Campus by an international, gender- and career-stage-mixed team. All authors meet the Nature Portfolio authorship criteria; roles and responsibilities were agreed in advance. The study involved no fieldwork, local partners in other countries, or transfer of biological materials or traditional knowledge. No issues of community risk, stigmatization, or benefit-sharing were implicated.

### Reporting summary

Further information on research design is available in the Nature Portfolio Reporting Summary linked to this article.

## Supplementary information

Supplementary Information

Reporting Summary

Transparent Peer Review file

## Data Availability

All data leading to the figures and results in the main text and supplementary material have been uploaded to the Zenodo repository: 10.5281/zenodo.18678181. Images are available within the figures themselves.

## Code availability

All code to reproduce the figures and results in the main text and supplementary material was written in Python or R and has been uploaded to the Zenodo repository: 10.5281/zenodo.18678181.
