# Supplementary material for: Generation of marmoset monkeys with a non-mosaic disruption of the OTOF gene as a model of human deafness
Source: Nat Commun. 2026 Mar 28;17:3033. doi: 10.1038/s41467-026-71047-1 (PMC13036021; doi:10.1038/s41467-026-71047-1)
Supplement: Supplementary file 1 — Supplementary Information [file 41467_2026_71047_MOESM1_ESM.pdf]

Supplementary Material

List of supplementary material:

1. Supplementary Figure 1: PCR fragment length analysis by capillary electrophoresis
2. Supplementary Figure 2: Genotyping results
3. Supplementary Figure 3: PCR genotyping of a sperm sample from Animal #8
4. Supplementary Table 1. Experiments performed with the optimized CRISPR/Cas9 mix injected into zygotes
5. Supplementary Material 1: Off-Target analysis of OTOF-KO animal #5.
6. Supplementary Material 2: Further statistical analysis of hearing tests.
7. Supplementary Figure 4. Analysis of putative ABR response of OTOF-KO animal #8
8. Supplementary Figure 5. ALFA-Tag immunofluorescence of the organ of Corti of AAV-treated animal 5

Supplementary Figure 1: PCR fragment length analysis by capillary electrophoresis

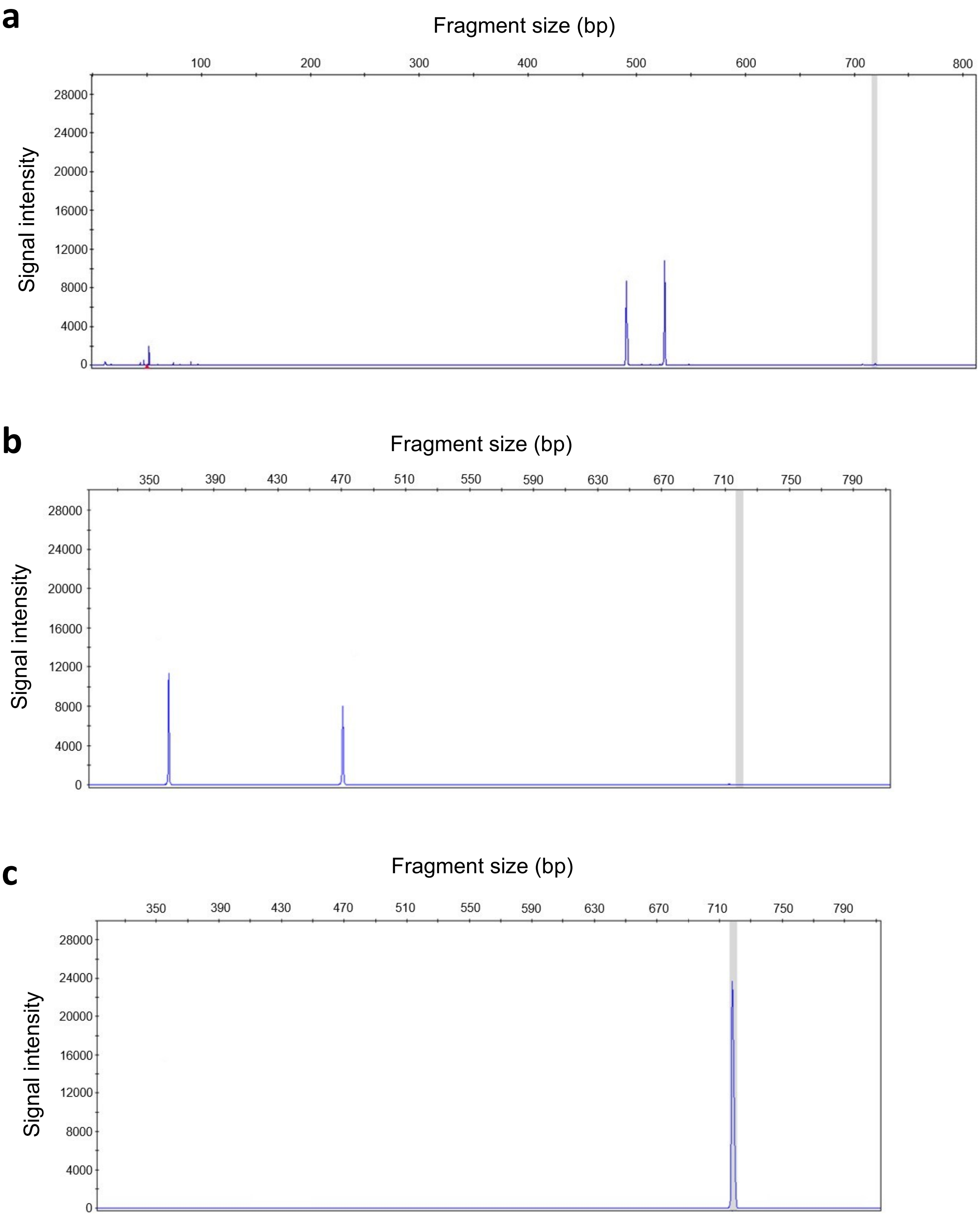

Supp. Fig. 1: PCR fragment length analysis by capillary electrophoresis. PCR was performed using primers 1 and 2, flanking the region containing the sgRNA binding sites. The grey bar indicates the wildtype (wt) fragment length. **(a)** PCR on DNA from buccal swabs of animal #5. Two fragments of 526 bp and 491 bp were detected. **(b)** PCR on DNA from buccal swabs of animal #8. Two fragments of 471 bp and 361 bp were detected. **(c)** PCR on wt DNA. The expected wt fragment length of 718 bp was detected.

Supplementary Figure 2: Genotyping results

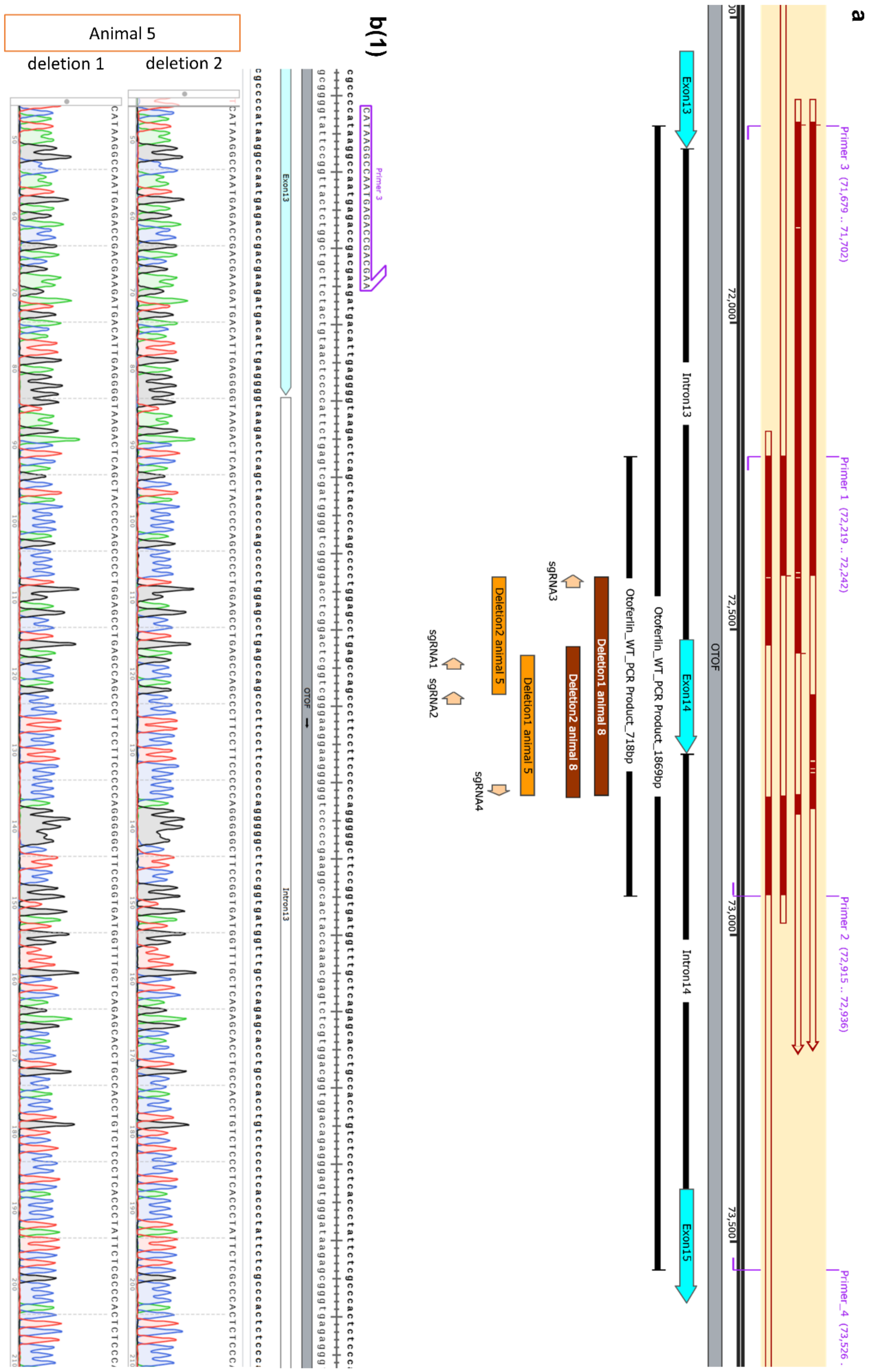

b(2)

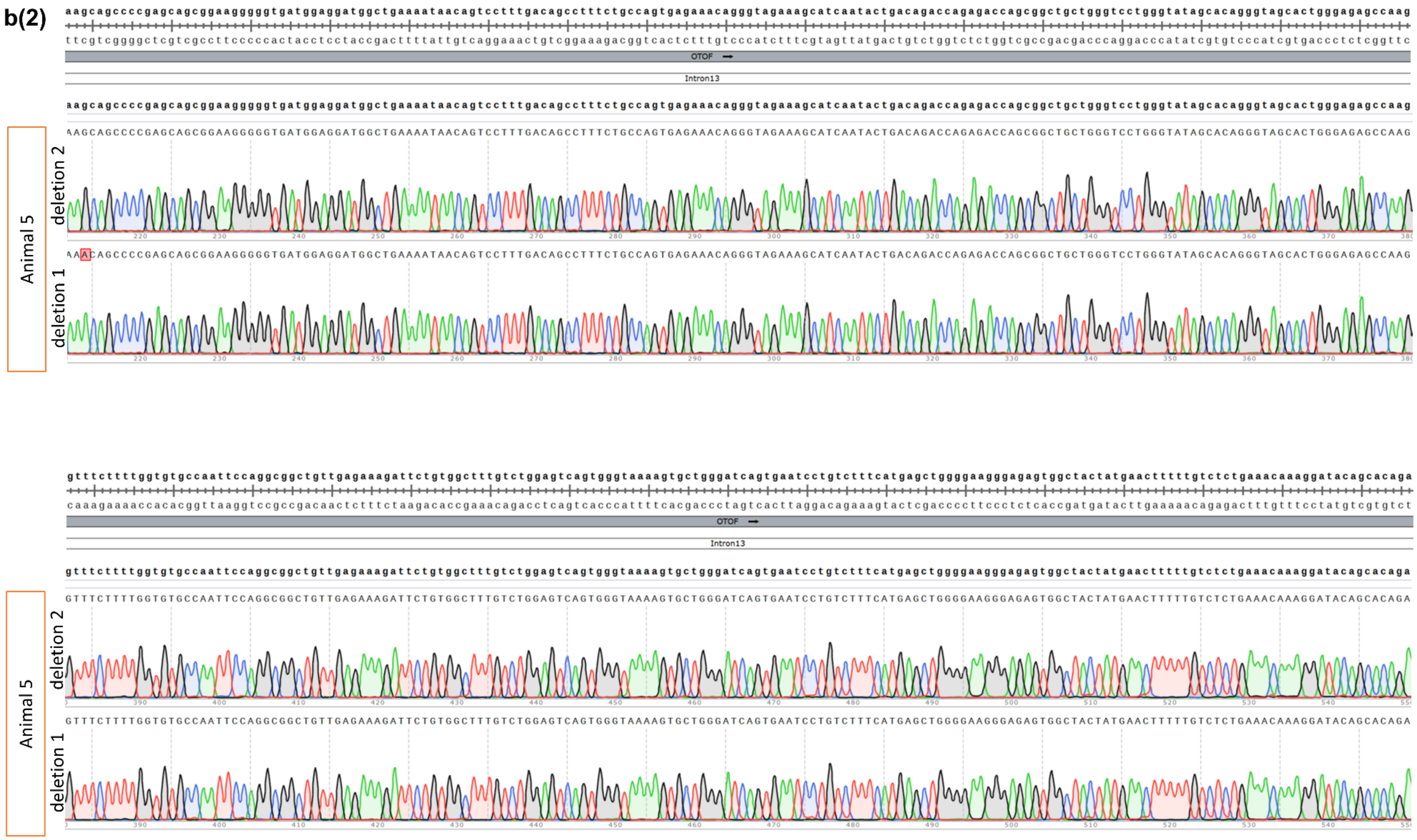
OTOF
Intron13
Animal 5
deletion 2
deletion 1
OTOF
Intron13
Animal 5
deletion 2
deletion 1

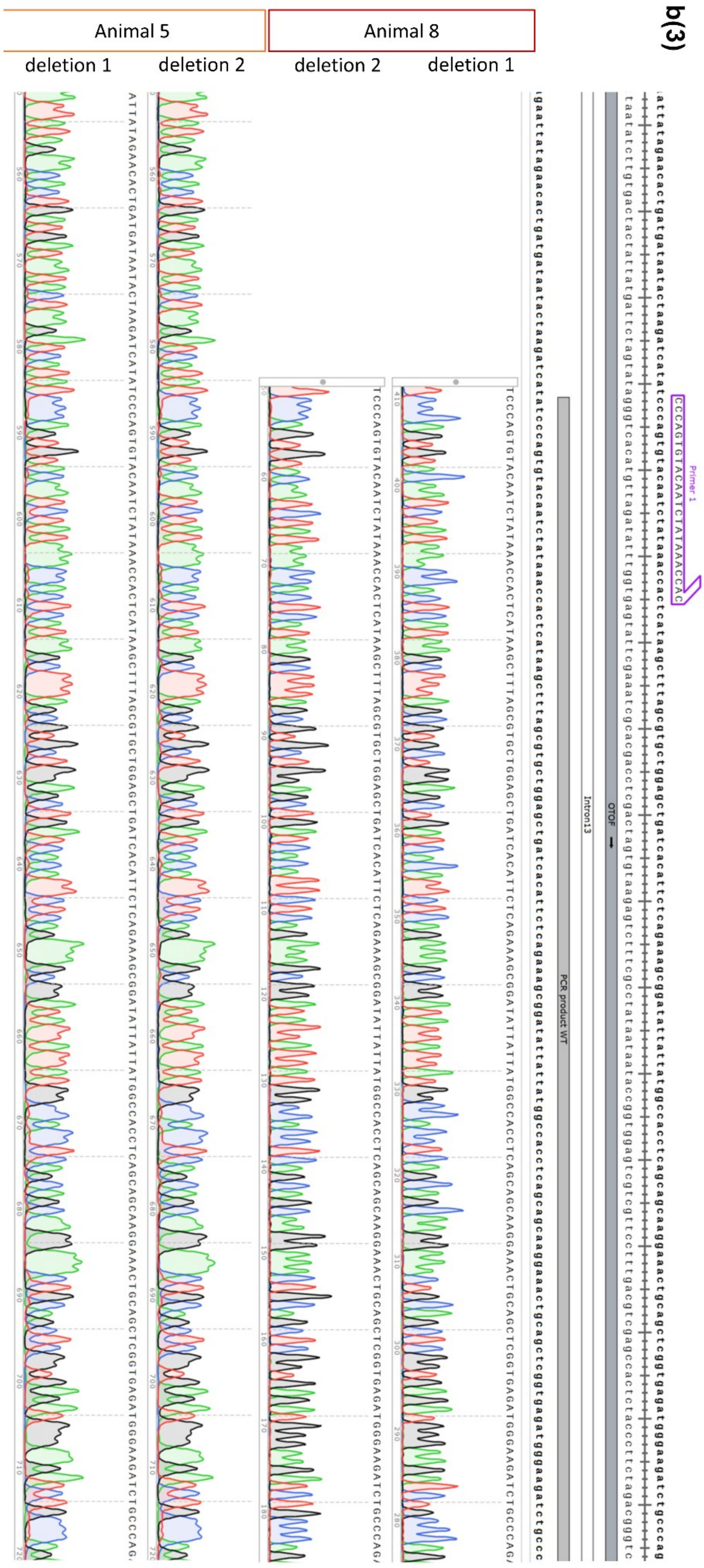
b(3)
Animal 5
Animal 8
deletion 1
deletion 2
deletion 2
deletion 1
Primer 1
OTOF
Intron13
PCR product WT

b(4)

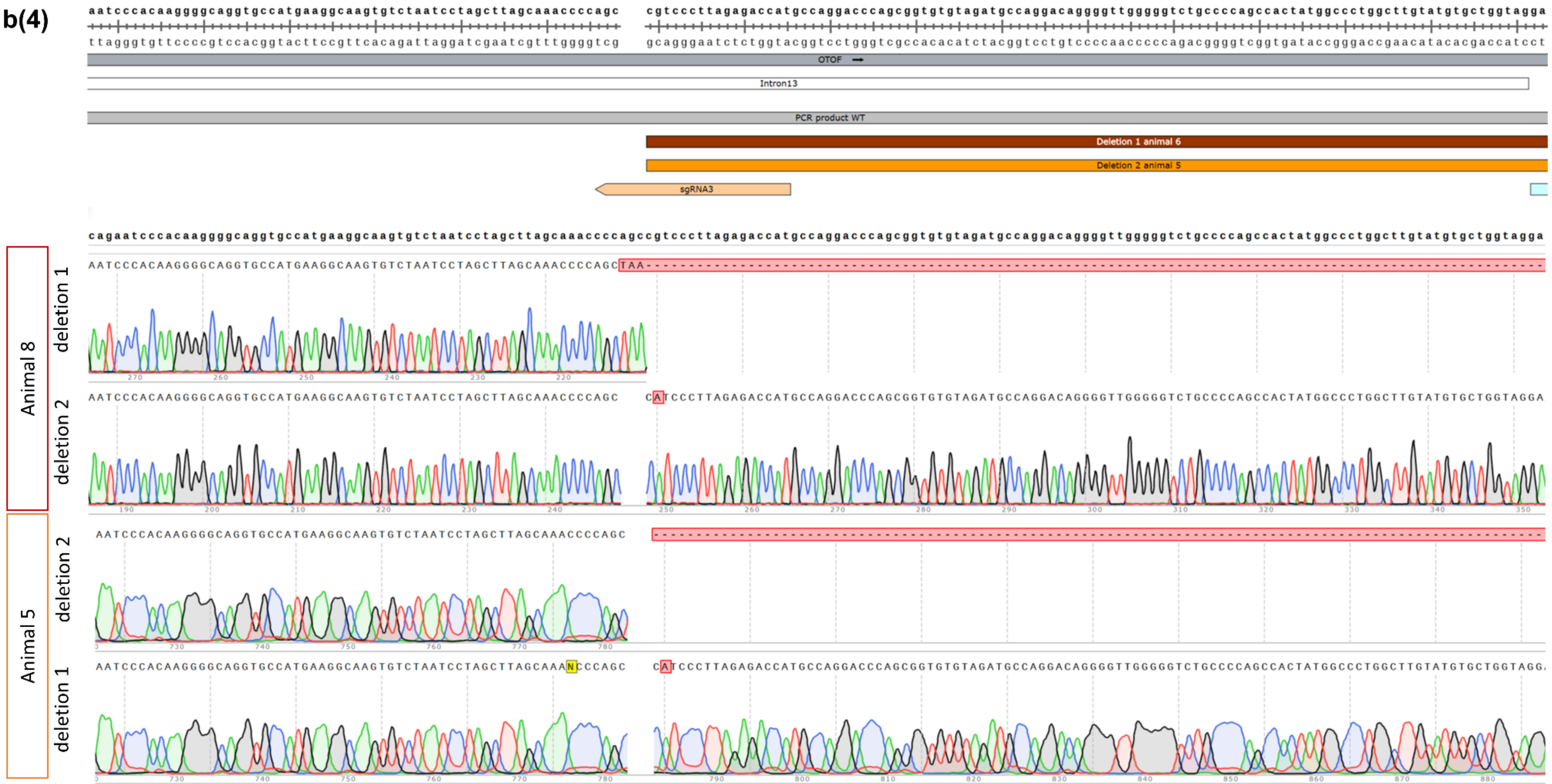

OTOF
Intron13
PCR product WT
Deletion 1 animal 6
Deletion 2 animal 5
sgRNA3
Animal 8
deletion 1
deletion 2
Animal 5
deletion 2
deletion 1

b(5)

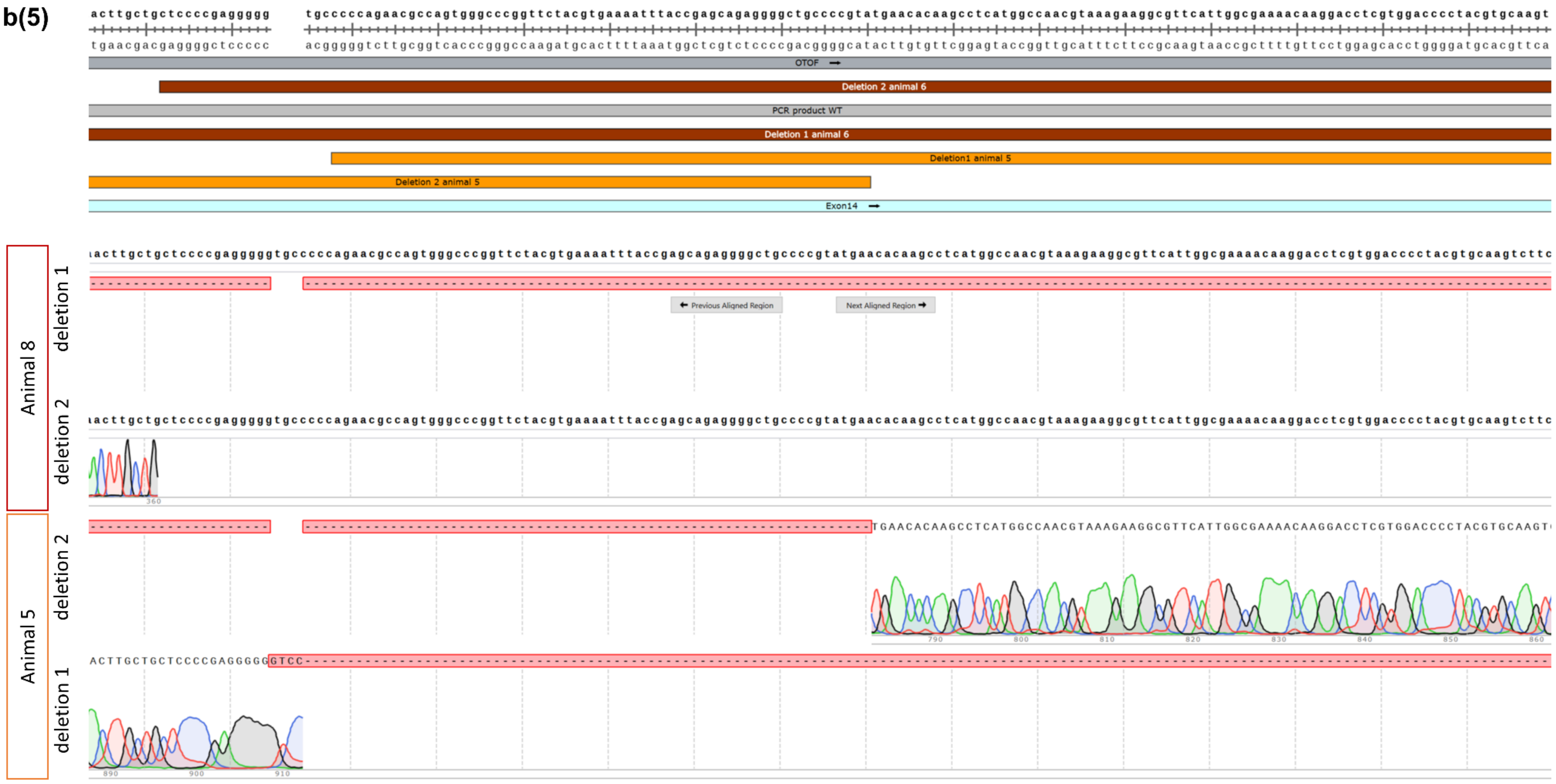

OTOF
Deletion 2 animal 6
PCR product WT
Deletion 1 animal 6
Deletion1 animal 5
Deletion 2 animal 5
Exon14
Animal 8
deletion 1
deletion 2
Previous Aligned Region
Next Aligned Region
Animal 5
deletion 2
deletion 1

b(6)

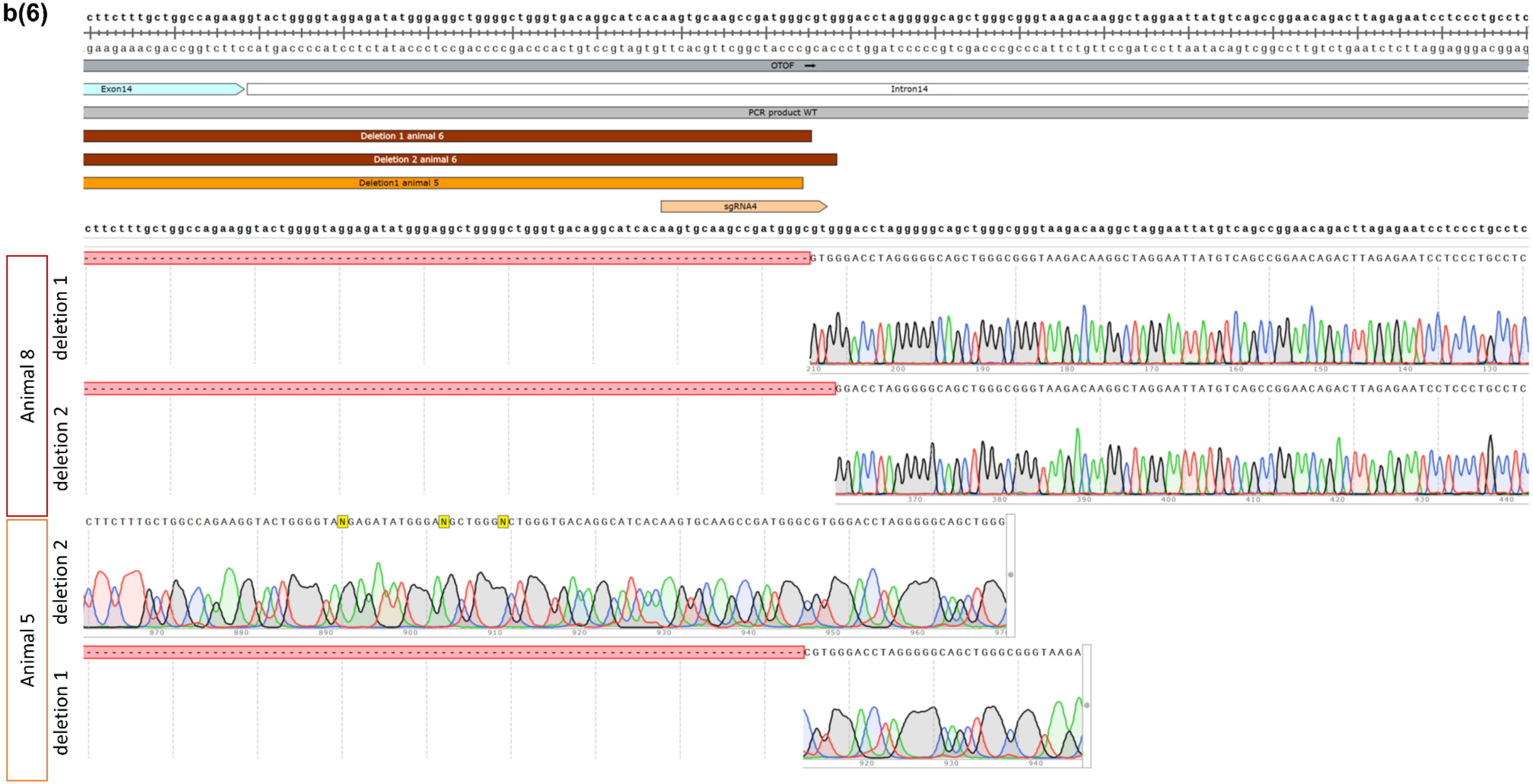

OTOF
Exon14
Intron14
PCR product WT
Deletion 1 animal 6
Deletion 2 animal 6
Deletion1 animal 5
sgRNA4
Animal 8
deletion 1
deletion 2
Animal 5
deletion 2
deletion 1

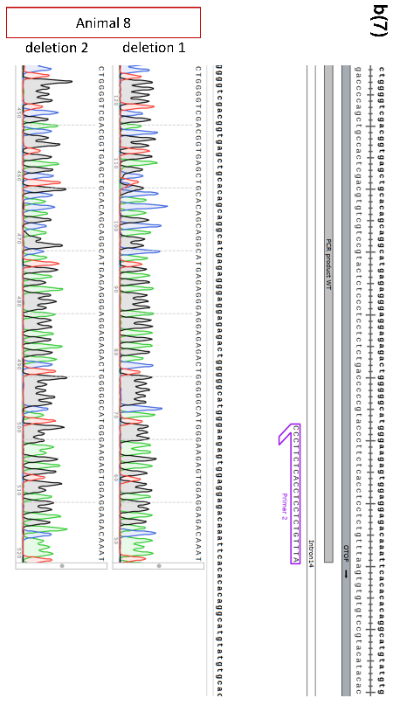

Supp. Fig. 2: Genotyping results of animals 5 and 8. PCR products derived from isolated genomic DNA of animals 5 and 8 using primers 3 (CATAAGGCCAATGAGACCGACGAA) and 4 (CACGTCGTTGACCTTGTCCGAG) were subcloned using TOPO cloning and sequenced. Sequences obtained were aligned to the *OTOF* gene of *C. jacchus* reference genome NC_071455.1 Chromosome 14, mCalJac1.2pat.X, INSDC Assembly GCF_011100555.1; *OTOF* gene ID: 10040869), displayed in grey. Exemplary aligned sequences of animal 5 (orange) and animal 8 (red) derived from DNA isolated from buccal swaps are shown. Deletions in the animals' alleles are indicated by light red boxes.

Supplementary Figure 3: PCR genotyping of a sperm sample from Animal #8

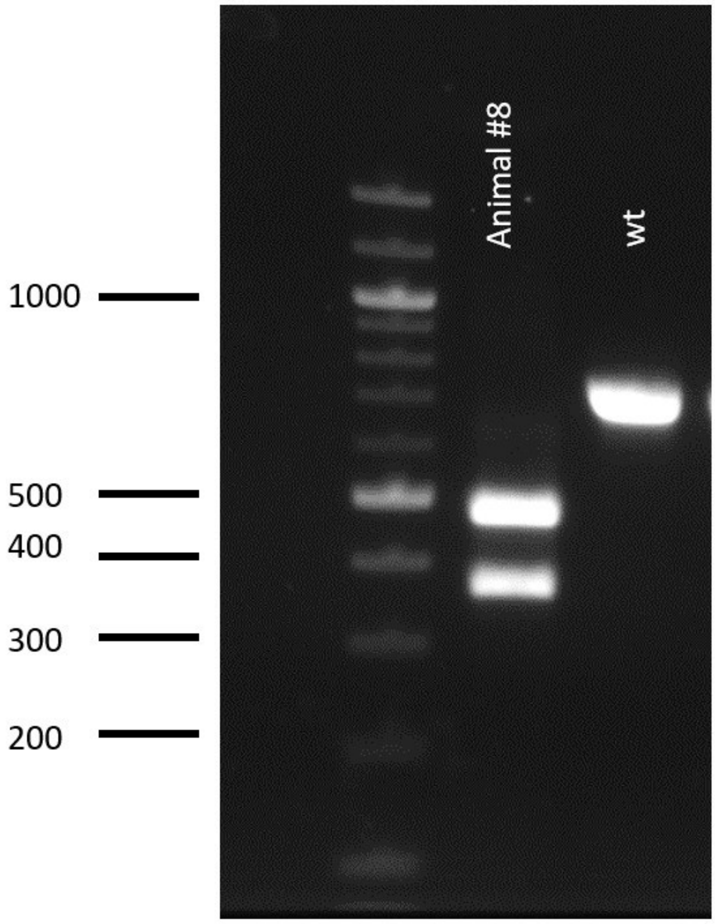

Suppl. Fig. 3: PCR genotyping of DNA samples from Animal #8 sperm (collected at 14 months of age and purified by swim-up) compared to marmoset wildtype (wt) sperm. Each PCR was performed with primers 1 and 2 flanking the region containing the sgRNA binding sites. The results are consistent with the PCR results for somatic cells from Animal #8 shown in Figure 2a and thus indicate the same genomic modification of the germ cells as occurred in somatic cells..

Supplementary Table 1. Experiments performed with the optimized CRISPR/Cas9 mix injected into zygotes

Additional protocol variables	OPU, n	Transfers, n	Embryos transferred, n	Newborns, n	Genetically modified newborns, n	Percentage of genetically modified newborns per transfer, %	Percentage of genetically modified newborns from all transferred embryos, %
Old anaesthesia	22 OPU	5	15	3	1	20%	6.7%
New anaesthesia + frozen transfers	20 OPU	15	41	1	1	7%	2.4%
Total	42	20	56	4	2	10%	3.6%

Supplementary Material 1: Off-Target analysis of OTOF-KO animal #5.

Suppl. Table 2: Primers and resulting PCR fragments in Off-Target analysis

**Name**	**Sequence**	**Chromo some**	**Expected Fragment size (bp)**
sense sgRNA1_offtarget1	5'-GTAGGGACTGCAGAGTGCACAA -3'	10	1155
antisense sgRNA1_offtarget1	5'-TGGCATTACCCAGTAAGTGCTCA -3'	10	
sense sgRNA1_offtarget2	5'-CAAGCATTTTCTATGTGCGGTCTG -3'	2	1324
antisense sgRNA2_offtarget2	5'-TTAGCCACCACATCCATAGGACA -3'	2	
sense sgRNA1_offtarget3	5'-TTCCTCTATGGCTCCCCTAACCC -3'	5	1138
antisense_ sgRNA1_offtarget3	5'-CCAGAATGCACCTCAGTGACC -3'	5	

sense sgRNA1_offtarget4	5'-GCCAGAGTATGCAGAACTGAC -3'	19	1124
antisense sgRNA1_offtarget4	5'-GAAACAGGTGAAATTCCTCGT -3'	19	
sense sgRNA2_offtarget1	5'-TCATTAGGCTCAAATCAAGGCATC -3'	X	1280
antisense sgRNA2_offtarget1	5'-GAGATTTATTTTGCCAAGGTTCAGG -3'	X	
sense sgRNA2_offtarget2	5'-CTTTGAAGTTAAGCATATAGCCCTC -3'	1	1222
antisense sgRNA2_offtarget2	5'-TGTAGTATCTGCACGTAATACTCACAC -3'	1	
sense sgRNA2_offtarget3	5'-TGAGTTACACTGGAGGCTTCAAC -3'	20	956
antisense sgRNA2_offtarget3	5'-CCAATTAGAAAAGAGCAACAGCAC -3'	20	
sense sgRNA2_offtarget4	5'-CTACAGTTTTCAGTGCCAACAGG -3'	1	959
antisense sgRNA2_offtarget4	5'-GCCCCAAAGTCAATGTATAGTCG -3'	1	
sense sgRNA3_offtarget1	5'-CCGTGATCAAACACTGCAAGGAC -3'	8	1393
antisense sgRNA3_offtarget1	5'-GCAGTACATCTCCGCAAGAGC -3'	8	
sense sgRNA3_offtarget2	5'-TACAAGTGCATGTAATGTCCACGTC -3'	5	1255
antisense sgRNA3_offtarget2	5'-TTCCTGCTTCTCATCAAACTTGCCTA -3'	5	
sense sgRNA3_offtarget3	5'-GAGAAGTTCTAGTAAATGCCAAGCC -3'	2	1190
antisense sgRNA3_offtarget3	5'-GTCACCAATTCCACCCATGCAC -3'	2	

sense sgRNA3_offtarget4_5	5'-TCAAAACTGGGGAGAGAGCAAGCTTC -3'	22	1393
antisense sgRNA3_offtarget4_5	5'-ACTTGTCCAAATGCAACAACAGCC -3'	22	
sense sgRNA4_offtarget1	5'-CACTCAGTTTCCATTTGGTGCTT -3'	12	1255
antisense sgRNA4_offtarget1	5'-CCCTCCCCACCACAACACTCT -3'	12	
sense sgRNA4_offtarget2	5'-GCTTTCTCCCAATTTACCTGCT -3'	1	1190
antisense sgRNA4_offtarget2	5'-GCCTCAAATCCCTGATGACCAC -3'	1	
sense sgRNA4_offtarget3	5'-TGCTATATATCAGTTAGGACGGCAAA -3'	10	1210
antisense sgRNA4_offtarget3	5'-TCAATGCGAAAGTTTGATTTAAGCTA -3'	10	
sense sgRNA4_offtarget4	5'-CTAAGTCTACCGGAAAGTATAGGC -3'	12	1119
antisense sgRNA4_offtarget4	5'-TAAACATCATGAATGGAGCTAAGCATC -3'	12	
antisense2 sgRNA4_offtarget1	5'-CAGCCAGAGACTGCACAATGTCA -3'	12	1155

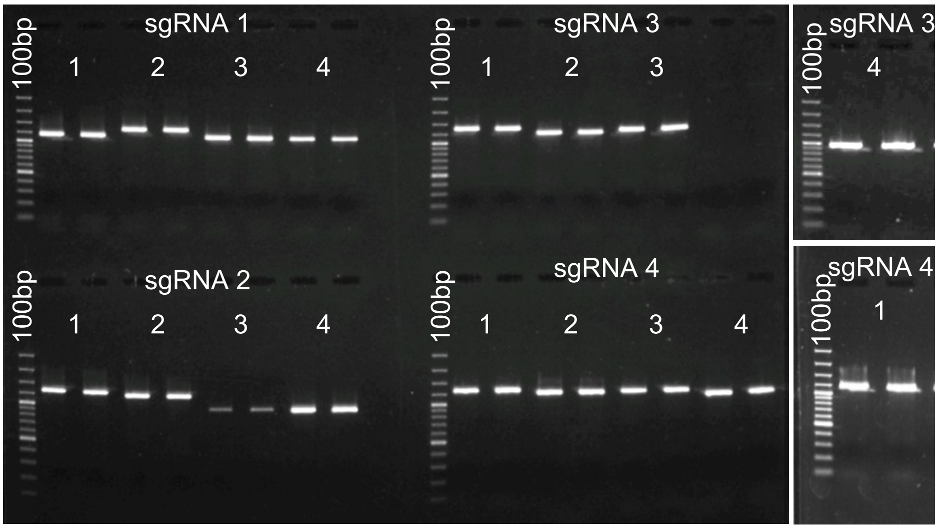

Suppl. Fig. 4: **Off-Target analysis of animal #5.** Visualization of PCR results for animal 5 by gel electrophoresis. Analysis for sgRNA3, target 4 and sgRNA4, target 1 were repeated separately (right) due to lack of product or unclear sequencing results, respectively.

1µl PCR product separated on a 1% agarose gel containing 0.25x GelRed (Biotium, VWR, #41003-1), 100bp size marker (Thermo Scientific 8ng/µl, #SM0321), PCR products were subsequently sequenced by Sanger sequencing, no off-target effects were detected.

Supplementary Material 2: Further statistical analysis of hearing tests

ABR:

In WT animals, ABR thresholds (Fig. 4B) were frequency dependent (repeated measures ANOVA: $F(4,24) = 6.76$, $p = 0.00086$). Post-hoc tests revealed significantly lower thresholds for 16 kHz (mean ± STD: 43.8 ± 9.16 dB SPL, n = 7 ears) in comparison with 2 kHz (paired t-test with Bonferroni correction: $p = 0.008$; mean ± STD = 57.1 ± 12.5 dB SPL, n = 7 ears) and 4 kHz (paired t-test with Bonferroni correction: $p = 0.041$; mean ± STD = 47.1 ± 9.51 dB SPL, n = 7 ears). For further statistical evaluation and if no response was observed, thresholds were set to 110 dB SPL. A repeated measures ANOVA with within subject factor frequency and between subject factor genotype revealed significantly different thresholds of KO and WT animals ($F(1,8) = 1008.14$, $p = 1.05*10^{-9}$). No main effect of frequency was observed but a significant interaction with genotype ($F(2.7,21.57) = 3.28$, $p = 0.045$). Post-hoc t-tests revealed that thresholds for KO animals were significantly higher than thresholds in WT animals for all pure tone frequencies (2 kHz, p = 2.12e-04; 4 kHz: p = 3.99e-06, 8 kHz: p = 4.99e-06, 16 kHz: p = 4.51e-08; each with $n_{wt} = 7$ ears, $n_{ko} = 3$ ears) as well as the click (p = 6.26e-08, $n_{wt} = 8$ ears, $n_{ko} = 4$ ears).

DPOAE:

A repeated measures ANOVA including within-subject factor frequency and between-subject factor genotype revealed a significant main effect of frequency ($F(5,10) = 8.614$, $p = 0.002$) but not of genotype ($F(1,2) = 1.086$, $p = 0.424$). Independent of genotype, thresholds for F1 = 13.9 kHz (mean ± STD = 51 ± 5.48 dB SPL, n = 5 ears) were significantly lower than for F1 = 2.4 kHz (mean ± STD = 82 ± 4.47 dB SPL, n = 5 ears) and F1 = 3.5 kHz (mean ± STD = 75 ± 0 dB SPL, n = 4 ears; post hoc t-tests with Holm correction: 2.4 kHz vs. 13.9 kHz $p \leq 0.0002$; 3.28 kHz vs. 13.9 kHz $p = 0.009$; Fig. 4D).

Supplementary Figure 5: Analysis of putative ABR response of OTOF-KO animal #8.

In animal 8, a response to stimulation with 2 kHz pure tones was observed at 100 dB SPL and higher. Similar responses were not observed in the second KO animal (animal 5; Suppl. Fig. 5a). ABR peaks were quantified with respect to their latency (Suppl. Fig. 5b) and amplitude (Suppl. Fig. 5c). The latencies of the waves of the 2 kHz response for KO animal 8 were comparable to ABR responses in WT animals. A two-way ANOVA with factors genotype and wave revealed a significant effect of wave ($F(5,37) = 133.968$, $p = 1.22*10^{-22}$) and a significant interaction between genotype and wave ($F(3,37) = 3.125$, $p = 0.037$). This indicates that the 2 kHz response for KO animal 8 differs from WT animals. The amplitudes increased much faster than expected from WT data. Here, a two-way ANOVA with factors genotype and wave revealed a significant effect of wave ($F(5,37) = 3.794$, $p = 0.007$) but also genotype ($F(1,37) = 14.807$, 2, $p = 0.000455$) and a significant interaction between genotype and wave ($F(3,37) = 4.94$, $p = 0.006$). We sought to further substantiate this observation by checking whether the waves can be considered outliers. Out of all detected waves, the amplitude of 2 waves were determined to be outliers and extreme. Both data points stem from animal 8 (KO, Suppl. Fig. 5c). Rosner's test for outliers revealed that 2 data points (R.1 = 3.66, R.2 = 4.08) are outliers and are not from the same distribution ($p < 0.05$). Taken together, the data indicates a potential pathological response at 2 kHz found for animal 8 (KO) and do not indicate the activation of the auditory pathway. Splitting the trials between phase and antiphase stimulation (Suppl. Fig. 5f) reveals a consistent waveform, arguing against a methodological artefact or cochlear microphonic potential and in favor of a physiological response.

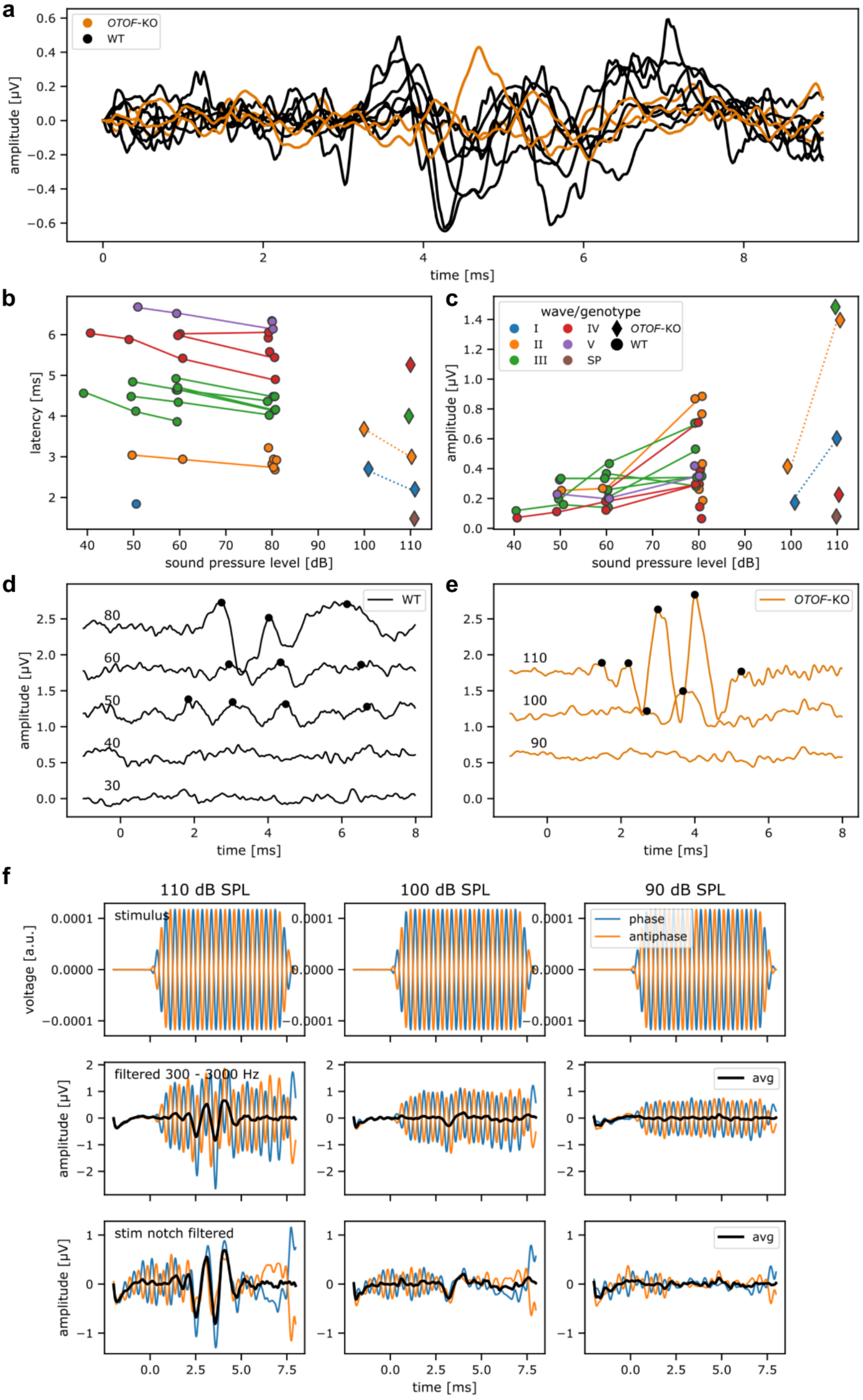

a
OTOF-KO
WT
amplitude [µV]
time [ms]
b
latency [ms]
sound pressure level [dB]
c
wave/genotype
I
II
III
IV
V
SP
OTOF-KO
WT
amplitude [µV]
sound pressure level [dB]
d
WT
80
60
50
40
30
amplitude [µV]
time [ms]
e
OTOF-KO
110
100
90
time [ms]
f
110 dB SPL
100 dB SPL
90 dB SPL
stimulus
phase
antiphase
voltage [a.u.]
filtered 300 - 3000 Hz
avg
amplitude [µV]
stim notch filtered
avg
amplitude [µV]
time [ms]

Suppl. Fig. 5: Analysis of putative ABR response of OTOF-KO animal 8. a) ABR measurements of WT (black) and *OTOF*-KO (orange) animals to 2 kHz stimulation at 80 dB SPL (WT) or 100 dB SPL (KO). Each wave corresponds to data from one ear. **b**) latencies and **c**) amplitudes of identified ABR peaks (nomenclature following Harada&Tokuriki [1]). Diamonds and dashed lines were used for KO data whereas circles and solid lines were used for WT data. 10 dB above threshold KO animal had high ABR peak amplitudes. Exemplary ABR growth functions of one WT (**d**) and KO (**e**) animal. The numbers on the left of a curve correspond to the sound level in dB (dB SPL). Identified peaks are given with black dots. **f**) data of animal 8 for 2 kHz stimulation averaged separately for trials with the stimulus presented either in phase (blue) or in antiphase (orange). Top row is the recorded stimulus and the filtered recorded data (between 300 Hz and 3 kHz) is on the second row. From left to right the sound level decreases by 10 dB. Using a 2kHz notch filter on the data is shown in the lower row. Based on this analysis the putative response occurs both in phase and antiphase.

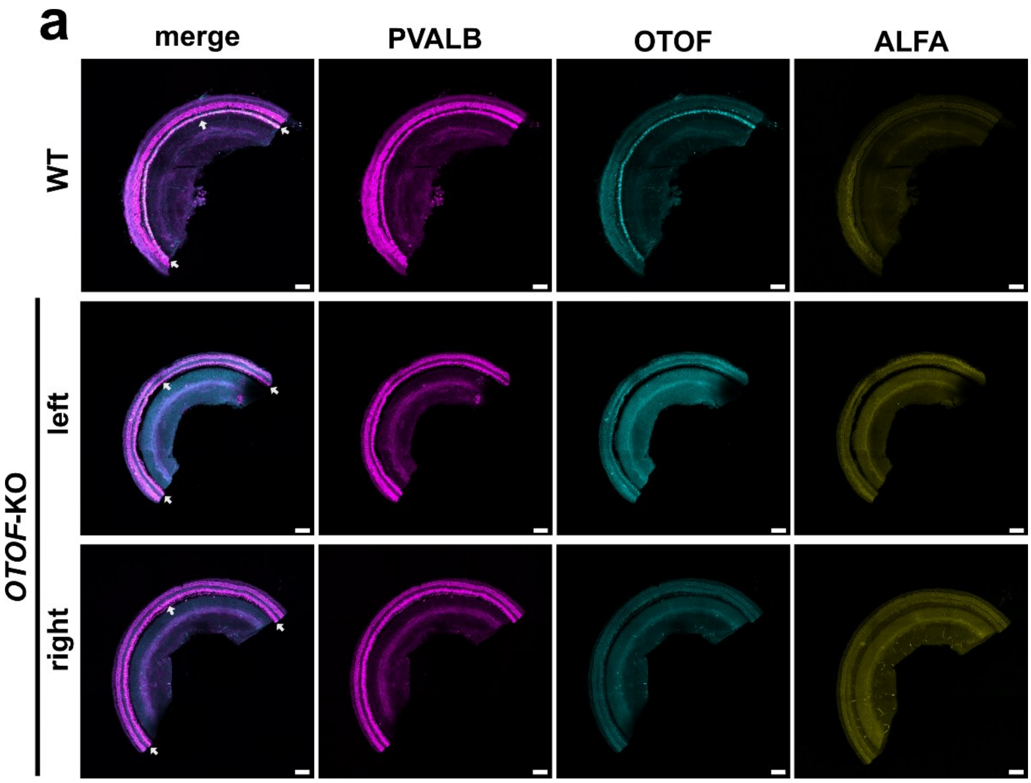

Suppl. Fig. 5: ALFA-Tag immunofluorescence of the organ of Corti of AAV-treated animal 5 (same images as in figure 7 for parvalbumin and otoferlin). Immunofluorescence for the ALFA-Tag present at the C-terminus of the *OTOF*-transgene administered via AAV into the left ear of animal 5 is depicted in yellow. No specific staining (above the background fluorescence level visible also in the non-treated control animal and the non-treated ear of animal 5) was detectable. Scale bar 100µm

1. Harada, T. & Tokuriki, M. Effects of click intensity and frequency on the brain-stem auditory evoked potentials in the common marmoset (Callithrix jacchus). *Journal of Veterinary Medical Science* **59**, 561–567 (1997).
