# Supplementary material for: Generation of marmoset monkeys with a non-mosaic disruption of the OTOF gene as a model of human deafness
Source: Nat Commun. 2026 Mar 28;17:3033. doi: 10.1038/s41467-026-71047-1 (PMC13036021; doi:10.1038/s41467-026-71047-1)
Supplement: Supplementary file 3 — Transparent Peer Review file [file 41467_2026_71047_MOESM3_ESM.pdf]

nature portfolio

Peer Review File

Generation of marmoset monkeys with a non-mosaic disruption of the OTOF gene as a model of human deafness

Corresponding Author: Dr Tobias Moser

This file contains all reviewer reports in order by version, followed by all author rebuttals in order by version.

Version 0:

Reviewer comments:

Reviewer #1

(Remarks to the Author)
The article titled "Generation of marmoset monkeys with a non-mosaic disruption of the OTOF gene as a model of human deafness" by Kahland describes the successful creation of a non-mosaic OTOF knockout marmoset model for DFNB9, a form of human genetic deafness. Using CRISPR-Cas9-mediated genome editing of embryos and zygotes, they obtain two homozygous OTOF-KO offspring that exhibit profound hearing loss and absent otoferlin expression in inner hair cells. Auditory brainstem response (ABR), distortion product otoacoustic emissions (DPOAE), and cochlear immunostaining analyses confirm auditory synaptopathy. The authors further characterize spontaneous vocalizations and suggest these are preserved in the absence of auditory feedback, providing a potential model for testing gene therapies or implants.

Major comment
1. The creation of a non-mosaic OTOF-KO marmoset model is a notable technical achievement and is thoroughly characterized, the absence of a therapeutic intervention (e.g., AAV-mediated OTOF gene delivery) represents a significant limitation. Given that human clinical trials are already underway and showing promising results, the main added value of this non-human primate model lies in its potential for translational validation, particularly regarding vector delivery, expression levels, and long-term functional recovery.
2. Only two KO animals were obtained from 81 embryo transfers. This low efficiency limits statistical interpretation and raises concerns about scalability.
3. Key challenges, low editing yield, high costs, ethical constraints, potential immune responses to Cas9 or gene therapy, are only briefly acknowledged.
4. No detailed discussion of how this model complements or surpasses rodent models in gene therapy applications.

Minor Points

1. Some figures (e.g., Figure 3 and 4) would benefit from clearer legends and labeling of axes. Indicate explicitly which data correspond to individual animals versus group means.
2. in Figure 5, include a higher-magnification inset of IHCs with and without otoferlin staining to better illustrate the immunofluorescence result.

Reviewer #2

(Remarks to the Author)
Results from independent Otoferlin (OTOF) gene therapy (GT) trials reported less than 2 years ago represent a breakthrough in hearing research. OTOF GT is a promising alternative to cochlear implant for pediatric DFNB9 patients. In addition, a recent report indicates that a 23-year-old DFNB9 patient also showed significant improvement in hearing after OTOF GT (Qi et al., The Lancet, 2025, Volume 405, Issue 10481, 777-779). Nevertheless, important questions related to OTOF-GT

including safety (immune response), durability, therapeutic window, and complete restoration of hearing remain to be addressed. Research using preclinical models is critical to advancing treatment to clinical trials and to patients. Hearing research has benefited enormously from studies in mice, which, among other things, helped advance GT trials for DFNB9. However, differences in anatomy, physiology, manifestation of the phenotype, vocal development, and lifespan create limitations to extrapolating results from mice to humans. Evaluating the efficacy of novel therapies in a non-human primate (NHP) genetic model for hearing loss is expected to yield results with greater translational predictive value compared to efficacy results from organoids and/or rodent models. NHP genetic models will be valuable in evaluating the efficacy, specificity, and durability of novel therapies. Kahland et al. report (manuscript under review) on the generation of marmoset monkeys with non-mosaic disruption of the OTOF gene as a model of human deafness. This is an important development that could help address preclinical optimization and characterization needs for OTOF GT. However, the small sample size – two OTOF KO marmosets – is not sufficient to support all the conclusions in the manuscript.

Main Comments:
1. A genetic modification protocol for NHPs that yield a low rate of mosaicism will be valuable for generating genetic models of hearing loss and other disorders. The authors used a multi-step protocol that combines IVF protocol in marmosets and CRISPR/Cas9 editing, in which each of the core methods used required fine-tuning or further optimization. The multistep protocol yielded two compound heterozygote OTOF KO offspring with hearing loss phenotype. The latter is consistent with the hearing loss phenotype previously reported in Otof KO mice, demonstrating successful targeting/disabling of OTOF in animals #5 and #6 (Fig. 1). However, the claim that they have developed an optimized multistep protocol to yield non-mosaic transgenic NHPs based on two KO animals (n=2) is concerning; additional KO animals with identical outcomes would support their claim. The challenges associated with generating a genetic model in a NHP species are recognized. However, the sample size (n=2 KOs) is too small to come to a firm conclusion.

2. Congenital deafness significantly impairs vocal development in DFNB9 patients due to the lack of auditory input. Slow and incomplete speech development in a DFNB9 patient who received OTOF GT would indicate limited efficacy of the specific AAV-OTOF gene sequence used or other novel therapy under investigation. In developing and optimizing novel therapies for DFNB9, our hope is that the development of vocalization in the OTOF KO NHP models could be a surrogate to address the GT efficacy issue. Intriguingly, in the 2 KO marmosets, the congenital deafness did not alter vocal pattern generation at the infant stage (2-4 weeks postnatal). The authors conclude, “This suggests that these vocalizations are a largely innate behavior and, at this level of analysis do not require hearing experience and auditory feedback” (lines 421-422). There is evidence in the literature (as the authors note) that auditory input/feedback is integral to the development/maturation of vocal pattern generation in marmosets. For example, their ability to produce mature-sounding contact calls. Did the authors record vocal patterns generated in the KOs beyond postnatal week four? It is interesting that the hearing status of the animals was assessed at 6 months of age, but the manuscript includes no indication that the vocal patterns of the KO animals were recorded beyond 4 weeks postnatal to document any deficiency in vocal development. For example, KO animals’ ability to produce mature-sounding contact calls compared to wild type controls?

3.Two KOs marmosets generated out of 56 viable embryos transferred to surrogates. This low yield (3.65%) will make it difficult to achieve a sufficient sample size for preclinical optimization and characterization of novel therapies, especially when there is wide variation in the treatment outcome. It is not clear if we can depend on breeding (in captivity) or would it be necessary to use the IVF-CRISPR/Cas9 protocol must be employed to generate sufficient number of OTOF KO animals to evaluate novel therapies. and allow for reproducibility of the protocol elsewhere. The authors should discuss and clarify this point in the manuscript. If we need to rely on the latter, that will further reinforce comment #1.

Other comments:
Line 147. Replace ‘regime’ with ‘regimen.’
Line 186: “ … was supplemented with 0-25 ng/µl hCas9 mRNA …” Why is this 0-25 ng/µl and not a fixed concentration, as it is for group 3? Please explain.
Also, it would appropriate to state ‘human codon optimized Cas9 nuclease’ before first use of the abbreviation ‘hCas9.’
Line 213: What about hCas9?
Lines 228-229: Could the authors elaborate on the refinement of the injection technique?
Line 348: Replace “ … produce…” with “produced …”

Reviewer #3

(Remarks to the Author)
This manuscript reports the successful generation of a non-human primate marmoset model of OTOF-related deafness. After gene therapy was performed in mice models for Otof-related deafness, clinical trials in humans have been done using AAV gene replacement. The transition from mice to humans has been rapid, with a need for exploring safety, efficacy and longevity in an intermediate model. Hence the authors created a CRISPR marmoset with an OTOF knock-out, replicating OTOF auditory synaptopathy. This CRISPR/Cas9-based editing strategy resulted in biallelic knockout animals that exhibit profound hearing loss.

In the introduction, the authors described the marmoset and its advantages as a non-human primate model (NHP). Moreover, they stated the ethical justification since hearing impairment is common and reduces quality of life. These marmosets will be relevant for explosring open questions regarding gene therapy treatment, including capsid-promotor combinations, an immunomodulation protocol and the consequences of treating both ears.

Guide RNAs were used to generate the editing of exon14 of the OTOF gene, with the modification of using 4 guides to avoid indels. A thorough description was provided regarding the creation of the marmoset knock-outs. Auditory synaptopathy was detected by using ABR and DPOAE. No otoferlin was detected in cochleas derived from one of the knock-out marmosets. No evidence was found for evidence for mosaicism, a common issue with marmosets.

Comments for improving the manuscript:

1. When does auditory function emerge in marmosets? Does it occur prenatally in the uterus as in humans, or postnatally as observed in mice? It would be worth adding these points to the introduction.
2. What is the similarity of the human and marmoset OTOF human orthologs? Please add this information.
3. The number of live births was quite low. Is this standard for marmoset or might there be an effect by OTOF on development?
4. The genotypes of two offspring are described. What is the genotype of the third live born animal (line 232)? It's not clear who animal 5 and 6 are relative to the 3 offspring that survived.
5. As the vocalization behavior was performed at the age of 2 to 4 weeks, and ABR at 6 months, it would be better to describe the results in chronological order.
6. At what age was the immunostaining performed? Were the images taken under the same conditions? It seems like there is a residual expression of OTOF, particularly in the left ear of the cochlea shown.
7. Did you check the hearing of the marmosets earlier prior to 6 months?

Reviewer #4

(Remarks to the Author)
The field of genetic forms of deafness has known considerable advances in the past 30 years. Since the characterization of the first deafness gene in the 1990s, there are currently more than 150 deafness genes that have been shown to cause isolated deafness and for which the underlying pathophysiological mechanisms have been or are being characterized. Since the 2010s, numerous preclinical attempts have shown that in several models, a substantial recovery of hearing can be achieved. In 2019, complete recovery of auditory thresholds after gene therapy could be demonstrated in mutant mice for the Otof genetic form. In 2024, several consortia around the world have injected the first vectors in patients and analysis of the degree of hearing recovery and its maintenance are under evaluation. The article by Tobias Kahland et al. reports the generation of marmoset monkeys with a non-mosaic disruption of the OTOF gene as a model of human deafness. The creation of a non human primate preclinical model is of importance because it will increase the safety of future generations of vectors rather than translating findings directly from mice to patients. The article is generally well written, clearly exposing the stakes of their work for the auditory field. It describes the challenges encountered to generate a stable model, characterizes the auditory phenotype of the OTOF model, and the nature of their vocalizations. Although this work is very important in terms of impact, a proof of principle on hearing restoration would have been a real plus, even if one has to admit that the long generation time of marmoset means that this work is likely to take several more years.

I have intermediate/minor issues that can in theory be addressed by the authors and are presented by order of appearance in the article :

-Introduction, line 65. The authors claim « some 1/1 human :mouse orthologs have developed new temporal and spatial expression trajectories.… ». Could the authors give precise examples of their claim ? It is generally considered that mice replicate very well the pathophysiology of deafness in humans with very rare exceptions.

-introduction line76. The claim « Clearly, a knowledge gap remains regarding the efficacy, specificity and longevity of gene therapy » is surprising coming from a group that contributes or has contributed to the otoferlin gene therapy and taking into account the fact that clinical trials are ongoing in patients. The authors should specify precisely based on articles which aspects need to be further examined carefully and why mice did not answer these questions. Otherwise, this sentence sounds as if enormous risks have been taken by translating findings in mice directly to patients. To the best of my knowledge, although an OTOF NHP model did not exist before, preclinical trias have tested toxicity and expression maintenance in NHPs.

-introduction , line 126. For the role of otoferlin, the authors must cite original papers. A key paper by Pangrsic et al, 2010 (PMID: 20562868) should be cited and the review is anterior to at least one other key paper like in 2017 (Michalski et al., PMID: 29111973). Together with these two papers, the original paper by Roux et al, 2006 should be recited.

-Results line 156 : What is special about exon 14 ? Could the authors mention to which otoferlin domain does it correspond and why the choice of that particular exon?

-Results : Have the authors tried to determine which Otof isoforms are expressed in the marmoset ? Several Otof isoforms have been reported in humans and mice. There are probably interspecies differences. It would be informative to know if the authors could establish whether there are several OTOF isoforms in marmosets.

-Results, line 335. Although I am a non specialist of this field, I respectfully disagree with the word « Intriguingly ». No other species comes close to humans in terms of language. It is probably the major specificity that differentiates us from other species. There has been several papers on mouse call in the past and the same result was found. There are no major

differences in the mouse vocalizations between hearing and deaf mice. The article would benefit from a discussion careflully comparing their results with similar studies in deaf mouse models. See for example : PMID: 23536072, PMID: 25062471

-Figure 3 : Marmoset WT ABRs seem to have 3 waves. However, in the supplementary figure, the authors label Waves1 to 5. Could the authors label the waves in their main figure and give wave details in terms of spieces differences between marmoset, humans and mice.

-Figure 5 : Figure 5 could benefit from bigger insets on IHCs. Why is there so much background in the OTOF KO marmoset compared to the WT control. Haven't the authors used the same settings in both conditions ? If not, this should be detailed.

-Discussion paragraph line 423-437. This paragraph is unclear. The authors did not find major differences in their call analysis but cite other articles that found differences in other experimental settings ; Could the author carefully report differences and comonalities with their study. Have the same developmental periods been compared ? how long were the parental deprivations etc…

-Discussion lines 450-454. The manuscript discussion should more indicate in much more details the reasons why a marmoset model is important for gene therapy trials and for cochlear implants.

-Discussion. I have not found any mention or comparison with other NHP models of deafness like for Usher syndrome. See PMID: 35710827

-Suppl Fig. 4. I am not sure to understand properly Suppl figure 4. It is somewhat misleading and gives the wrong impression of the importance of the unexplained waves in animal 6. These waves were obtained at important sound levels compared to wild-type controls. Therefore using a comparison based on hearing threshold gives these waves more unnecessary importance than needed. For instance, in Fig.1C, the panel gives the impression KO waves are 4 times bigger than in the WT just because there is a larger working range in WT mice. The authors should at least show what happens at higher levels than +30dB above threshold in WT monkeys. In panel « a », the comparison of a 80 dB signal in WT versus a 100 dB signal in KO is also misleading. Between 80 and 100 dB, there is roughly a 100 fold difference in sound intensity…so putting in parallel these 2 signals that have similar amplitude are misleading. They should put in parallel the KO signal at 80 dB for example.

Minor :

-Abstract, line 42. The authors should tell why preclinical optimization remains invaluable to establish OTOF-gene therapy.

-Results line 308 : Typo on « A repeated measures ANOVA with within subject… » ?

-line 653. There is a typo « a94% »

- I am uncertain there is a Supplementary figure 2 legend on genotyping results

-line 43. Typo at « Fig (XB) » ?

Version 1:

Reviewer comments:

Reviewer #1

(Remarks to the Author)
The authors have addressed the reviewer comments thoroughly and thoughtfully. The additional clarifications and revisions have markedly improved the clarity and scientific rigor of the manuscript. I particularly appreciate the expanded discussion highlighting the translational and late-preclinical relevance of the OTOF-KO marmoset model. The newly added section clearly delineates its potential applications for AAV-based OTOF gene therapy and its implications for cochlear implant research. Although direct experimental validation of gene delivery falls outside the scope of this study, the authors have appropriately acknowledged this limitation.

Reviewer #2

(Remarks to the Author)
The detailed responses to comments and changes to manuscript are noted and appreciated. In the revised manuscript, a deviation from the WT vocal development was documented in the two KOs at later stages. This is interesting. However, it is not clear whether the marmoset Otoferlin KOs will mimic vocal development deficits observed in DFNB9 patients and, in that respect, serve as a superior surrogate to the mouse model for testing the efficacy of OTOF gene therapy. Nevertheless, the diligent work of the authors to optimize various steps in the protocol to improve the odds of generating non-mosaic disruption of OTOF and a model for deafness in DFNB9, are noteworthy. I believe this work will serve as an important reference for investigators hoping to be generating NHP genetic models. The manuscript's overall significance outweighs my reservations.

Sincerely,
Kumar

Reviewer #4

(Remarks to the Author)
The analysis of call duraation is a real plus for the study. The authors have generally well addressed all my questions and concerns except one minor:

-Despite announcing having added all key original papers, the authors omitted PMID: 29111973.
See comment in the first submission:
“Pangrsic et al, 2010 (PMID: 20562868) should be cited and the review is anterior to at least one other key paper like in 2017 (Michalski et al., PMID: 29111973). Together with these two papers, the original paper by Roux et al, 2006 should be recited.”

Open Access This Peer Review File is licensed under a Creative Commons Attribution 4.0 International License, which permits use, sharing, adaptation, distribution and reproduction in any medium or format, as long as you give appropriate credit to the original author(s) and the source, provide a link to the Creative Commons license, and indicate if changes were made.
In cases where reviewers are anonymous, credit should be given to 'Anonymous Referee' and the source.
The images or other third party material in this Peer Review File are included in the article’s Creative Commons license, unless indicated otherwise in a credit line to the material. If material is not included in the article’s Creative Commons license and your intended use is not permitted by statutory regulation or exceeds the permitted use, you will need to obtain permission directly from the copyright holder.
To view a copy of this license, visit https://creativecommons.org/licenses/by/4.0/

REVIEWER COMMENTS

Reviewer #1 (Remarks to the Author)

The article titled "Generation of marmoset monkeys with a non-mosaic disruption of the OTOF gene as a model of human deafness" by Kahland describes the successful creation of a non-mosaic OTOF knockout marmoset model for DFNB9, a form of human genetic deafness. Using CRISPR-Cas9-mediated genome editing of embryos and zygotes, they obtain two homozygous OTOF-KO offspring that exhibit profound hearing loss and absent otoferlin expression in inner hair cells. Auditory brainstem response (ABR), distortion product otoacoustic emissions (DPOAE), and cochlear immunostaining analyses confirm auditory synaptopathy. The authors further characterize spontaneous vocalizations and suggest these are preserved in the absence of auditory feedback, providing a potential model for testing gene therapies or implants.

We thank this reviewer for the comments and advice to further improve our manuscript. We have completely overhauled the MS following the reviewer's advice, and performed additional experiments and analyses, which further strengthened our manuscript. Below is our point-by-point response to the reviewer's concerns and comments.

Major comment

1. The creation of a non-mosaic OTOF-KO marmoset model is a notable technical achievement and is thoroughly characterized, the absence of a therapeutic intervention (e.g., AAV-mediated OTOF gene delivery) represents a significant limitation. Given that human clinical trials are already underway and showing promising results, the main added value of this non-human primate model lies in its potential for translational validation, particularly regarding vector delivery, expression levels, and long-term functional recovery.

We agree with the reviewer's assessment of the contribution regarding the methodological advance and the value for late preclinical work. In response to this comment, we have amended the discussion to better delineate the future use of the newly generated NHP model (last section of discussion).

2. Only two KO animals were obtained from 81 embryo transfers. This low efficiency limits statistical interpretation and raises concerns about scalability.

We achieved a major gain in efficiency after optimization of CRISPR activity, injection timing and technique, and optimization of embryo transfer, including more efficient synchronisation between the embryo recipient and the developmental stage of the in-vitro-produced embryo. The two genetically modified animals were obtained only after optimization of the injection mixture and the injection stage. We report all results, including those from the initial optimization experiments. Such data are rarely included in final manuscripts, but we consider them essential to prevent unnecessary trials by other groups and, hence and foremost, to reduce animal use in future studies.

After optimization of the injection mixture and stage, we performed five embryo transfers with a total of 15 embryos. Three animals were born (implantation rate 20%; 3/15), of which one was genetically modified. This corresponds to a rate of 20% genetically modified animals per transfer (1/5) and 6.7% per embryo transferred (1/15). Compared with outcomes reported by other groups working on genetic editing in nonhuman primates (reviewed in ref. 1), our results are comparable to or exceed most published values.

In a subsequent experimental phase, we adopted a new anesthesia regimen and, in some cases, transferred frozen–thawed embryos. The impact of each factor on embryo production and

implantation will be analyzed in future work and reported separately. Overall, during this phase the yield of genetically modified offspring declined (see Supplementary Table 1) but remained within the range reported for nonhuman primates, supporting the promise of our approach.

For clarity, outcomes obtained after protocol optimization are summarized in Supplementary Table 1.

Moreover, in response to the reviewer's comment, we have added the following statement to the section of the discussion that deals with limitations of our study: "Only two *OTOF*-KO animals were obtained from 81 embryos transferred in this present study. We achieved a major gain in efficiency from learnings such as the success with increased CRISPR activity. Our most efficient protocol resulted in rates of 20% genetically modified animals per transfer and of ~7% per transferred embryo (Supplementary Table 1), which is comparable to or exceeds most published values[1]. Future work will generate further animals using the optimized protocol and by cross-breeding the existing *OTOF*-KOs with wildtype marmosets. Parallel efforts of generating *OTOF*-mutant macaques from a spontaneously arisen mutation will help serving the urgent needs described above[2]. This notwithstanding, the generation times will remain a limiting determinant for the availability of the NHP models of DFNB9."

3. Key challenges, low editing yield, high costs, ethical constraints, potential immune responses to Cas9 or gene therapy, are only briefly acknowledged.

In response to the reviewer's comment, we extended the discussion accordingly: "Further challenges of our approach concern the risk of off-target CRISPR effects. We consider it low due to the short lifetime of CAS9-protein, *hCas9* mRNA, and sgRNAs as compared to plasmid-encoded *hCas9* and plasmid encoded sgRNAs. Moreover, the Cutting Frequency Determination (CDF) specificity score was above 90 out of 100 for all four guide RNAs, indicating a very low probability for off-target effects[3,4], and the 16 bioinformatically most likely off-target sites[75,76] were found to be unaltered (Suppl. Mat. 1)."

4. No detailed discussion of how this model complements or surpasses rodent models in gene therapy applications.

In response to the reviewer's comment, we extended the discussion accordingly (Value of OTOF-KO marmosets for future work and limitations of the present study): "Here, *OTOF*-KO NHP models could play a critical role in improving *OTOF*-related gene therapy before moving to clinical trials in humans, complementing the highly relevant work in mouse mutants. For example, NHP models will allow for extended studies on efficacy and longevity of functional restoration upon gene therapy. Correlating auditory function following *OTOF*-related gene therapy assessed at the physiological and behavioral level with postmortem analysis of otoferlin expression in IHCs will allow to optimize dosing, vectors, promotors, transgene, and AAV administration. Moreover, NHP models will support late-preclinical studies of the dependence of the functional outcome on time point of intervention, AAV-dose, presence of neutralizing antibodies, and immunomodulation, and will allow analyses of off-target expression. In addition, *OTOF*-KO marmosets could be useful for the development of next-generation cochlear implants. Further, this model uniquely allows to explore how auditory feedback shapes vocal development, thereby deepening our understanding of basic neuroscience and driving translational clinical research into auditory disorders. We conclude that the key role of appropriate NHP models of genetic deafness for late-preclinical testing of novel therapies offset the challenges, such as slow generation time, risk of off-target effects of CRISPR, potential immune responses to Cas9, high costs, and ethical concerns regarding disease modeling in NHP."

Minor Points

1. Some figures (e.g., Figure 3 and 4) would benefit from clearer legends and labeling of axes. Indicate explicitly which data correspond to individual animals versus group means.

This has been rectified.

2. in Figure 5, include a higher-magnification inset of IHCs with and without otoferlin staining to better illustrate the immunofluorescence result.

This has been rectified.

Reviewer #2 (Remarks to the Author)

Results from independent Otoferlin (OTOF) gene therapy (GT) trials reported less than 2 years ago represent a breakthrough in hearing research. OTOF GT is a promising alternative to cochlear implant for pediatric DFNB9 patients. In addition, a recent report indicates that a 23-year-old DFNB9 patient also showed significant improvement in hearing after OTOF GT (Qi et al., The Lancet, 2025, Volume 405, Issue 10481, 777-779). Nevertheless, important questions related to OTOF-GT including safety (immune response), durability, therapeutic window, and complete restoration of hearing remain to be addressed. Research using preclinical models is critical to advancing treatment to clinical trials and to patients. Hearing research has benefited enormously from studies in mice, which, among other things, helped advance GT trials for DFNB9. However, differences in anatomy, physiology, manifestation of the phenotype, vocal development, and lifespan create limitations to extrapolating results from mice to humans. Evaluating the efficacy of novel therapies in a non-human primate (NHP) genetic model for hearing loss is expected to yield results with greater translational predictive value compared to efficacy results from organoids and/or rodent models. NHP genetic models will be valuable in evaluating the efficacy, specificity, and durability of novel therapies. Kahland et al. report (manuscript under review) on the generation of marmoset monkeys with non-mosaic disruption of the OTOF gene as a model of human deafness. This is an important development that could help address preclinical optimization and characterization needs for OTOF GT. However, the small sample size – two OTOF KO marmosets – is not sufficient to support all the conclusions in the manuscript.

We thank this reviewer for the comments and advice to further improve our manuscript. We have completely overhauled the MS following the reviewer's advice, and performed additional experiments and analyses, which further strengthened our manuscript. Below is our point-by-point response to the reviewer's concerns and comments.

Main Comments:

1. A genetic modification protocol for NHPs that yield a low rate of mosaicism will be valuable for generating genetic models of hearing loss and other disorders. The authors used a multi-step protocol that combines IVF protocol in marmosets and CRISPR/Cas9 editing, in which each of the core methods used required fine-tuning or further optimization. The multistep protocol yielded two compound heterozygote OTOF KO offspring with hearing loss phenotype. The latter is consistent with the hearing loss phenotype previously reported in Otof KO mice, demonstrating successful targeting/disabling of OTOF in animals #5 and #6 (Fig. 1). However, the claim that they have developed an optimized multistep protocol to yield non-mosaic transgenic NHPs based on two KO animals (n=2) is concerning; additional KO animals with identical outcomes would support their claim. The challenges associated with generating a genetic model in a NHP species are recognized. However, the sample size (n=2 KOs) is too small to come to a firm conclusion.

We concur with the reviewer on the proof-of-concept nature of our study. We achieved a major gain in efficiency after optimization of CRISPR activity, injection timing and technique, and optimization of embryo transfer including more efficient synchronisation between the embryo recipient and the developmental stage of the in-vitro-produced embryo. The two genetically modified animals were obtained only after optimization of the injection mixture and the injection stage. We report all results, including those from the initial optimization experiments. Such data are rarely included in final manuscripts, but we consider them essential to prevent unnecessary trials by other groups and, hence and foremost, to reduce animal use in future studies.

After optimization of the injection mixture and stage, we performed five embryo transfers with a total of 15 embryos. Three animals were born (implantation rate 20%; 3/15), of which one was genetically

modified. This corresponds to a rate of 20% genetically modified animals per transfer (1/5) and 6.7% per embryo transferred (1/15). Compared with outcomes reported by other groups working on genetic editing in nonhuman primates (reviewed in ref. 1), our results are comparable to or exceed most published values.

In a subsequent experimental phase, we adopted a new anesthesia regimen. The impact of each factor on embryo production and implantation will be analyzed in future work and reported separately. Overall, during this phase the yield of genetically modified offspring declined (see Supplementary Table 1) but remained within the range reported for nonhuman primates, supporting the promise of the proposed approach.

For clarity, the outcomes obtained after protocol optimization are summarized in Supplementary Table 1.

In response to the reviewer's comment, we have now extended the last section of the discussion to cover the "Value of OTOF-KO marmosets for future work and limitations of the present study".

"Only two *OTOF*-KO animals were obtained from 81 embryos transferred in this present study. We achieved a major gain in efficiency from learnings such as on the failure of grafting genotyped embryos and the success with increased CRISPR activity. Our most efficient protocol resulted in rates of 20% genetically modified animals per transfer and of ~7% per transferred embryo (Supplementary Table 1), which is comparable to or exceeds most published values[1]. Future work will generate further animals using the optimized protocol and by cross-breeding the existing *OTOF*-KOs with wildtype marmosets. Parallel efforts of generating *OTOF*-mutant macaques from a spontaneously arisen mutation will help serving the urgent needs described above[2]. This notwithstanding, the generation times will remain a limiting determinant for the availability of the NHP models of DFNB9."

2. Congenital deafness significantly impairs vocal development in DFNB9 patients due to the lack of auditory input. Slow and incomplete speech development in a DFNB9 patient who received OTOF GT would indicate limited efficacy of the specific AAV-OTOF gene sequence used or other novel therapy under investigation. In developing and optimizing novel therapies for DFNB9, our hope is that the development of vocalization in the OTOF KO NHP models could be a surrogate to address the GT efficacy issue. Intriguingly, in the 2 KO marmosets, the congenital deafness did not alter vocal pattern generation at the infant stage (2-4 weeks postnatal). The authors conclude, "This suggests that these vocalizations are a largely innate behavior and, at this level of analysis do not require hearing experience and auditory feedback" (lines 421-422). There is evidence in the literature (as the authors note) that auditory input/feedback is integral to the development/maturation of vocal pattern generation in marmosets. For example, their ability to produce mature-sounding contact calls. Did the authors record vocal patterns generated in the KOs beyond postnatal week four? It is interesting that the hearing status of the animals was assessed at 6 months of age, but the manuscript includes no indication that the vocal patterns of the KO animals were recorded beyond 4 weeks postnatal to document any deficiency in vocal development. For example, KO animals' ability to produce mature-sounding contact calls compared to wild type controls?

We fully agree with the reviewer that a detailed comparative analysis of the vocal behaviour of KO and WT animals would be highly valuable and informative. This conviction drove us to design our experiments to be as similar as possible to earlier work by the lab of Steffen Hage (Gultekin et al. 2021; doi: 10.1126/sciadv.abf2938) in order to be able to compare the vocal development of KO and WT animals. Consequently, we did assess the vocal behaviour of our KO animals over time. For the resubmission, we have now included comparison of WT and KO vocal behaviour regarding the duration of vocalizations in 2 recording periods (week 2 – 4 and week 18 – 20, referring to data of (Gultekin et

al. 2021) for WT) illustrated in the new figure 5. Intriguingly, our analysis points to alternative developmental trajectories in KO vs. WT animals in that KO animals exhibit shorter maximum call durations and that their increase with age does not catch up with WT animals.
Currently, we feel that we do not have sufficient data to draw strong conclusions regarding developmental trajectories and similarities between mature sounding calls of WT and KO animals. For the original submission, we therefore chose to limit the analysis to rather coarse level of analysis.

3. Two KOs marmosets generated out of 56 viable embryos transferred to surrogates. This low yield (3.65%) will make it difficult to achieve a sufficient sample size for preclinical optimization and characterization of novel therapies, especially when there is wide variation in the treatment outcome. It is not clear if we can depend on breeding (in captivity) or would it be necessary to use the IVF-CRISPR/Cas9 protocol must be employed to generate sufficient number of OTOF KO animals to evaluate novel therapies. and allow for reproducibility of the protocol elsewhere. The authors should discuss and clarify this point in the manuscript. If we need to rely on the latter, that will further reinforce comment #1.

While we achieved a major gain in efficiency in the generation of *OTOF*-KOs we have extended the "limitations of the present study" section of the discussion in response to the reviewer's comment: "Future work will generate further animals using the optimized protocol and by cross-breeding the existing *OTOF*-KOs with wildtype marmosets. Parallel efforts of generating *OTOF*-mutant macaques from a spontaneously arisen mutation will help serving the urgent needs described above[2]. This notwithstanding, the generation times will remain a limiting determinant for the availability of the NHP models of DFNB9."

Other comments:

Line 147. Replace 'regime' with 'regimen.'

This has been rectified.

Line 186: " … was supplemented with 0-25 ng/µl hCas9 mRNA …" Why is this 0-25 ng/µl and not a fixed concentration, as it is for group 3? Please explain.

At the initial stage of the project, we determined that injection into MII oocytes was too traumatic and the 2-pronuclei stage is to be preferred for CRISPR/Cas9 mixture injection. We also continued to optimize the composition of the injection mixture. Group 2 included injections into zygotes performed with 15-20 ng/µl Cas9 protein and 10 ng/µl of each sgRNA. Some mixtures injected into Group 2 zygotes were additionally supplemented with 25 ng/µl hCas9 mRNA. Since none of the 3 embryos developed after injection with mRNA has been transferred, we did not include those experiments into a separate group. For optimal clarity, we changed the text accordingly.

Also, it would appropriate to state 'human codon optimized Cas9 nuclease' before first use of the abbreviation 'hCas9.'

This has been rectified.

Line 213: What about hCas9?

This has been rectified.

Lines 228-229: Could the authors elaborate on the refinement of the injection technique?

Under “refinement of the injection technique” we mean fine-tuning of the injection technique with optimization of the microinjector flow rate, speed of needle insertion and withdrawal, depth of needle insertion, etc.

Line 348: Replace “ … produce…” with “produced …”

This has been rectified.

Reviewer #3 (Remarks to the Author):

This manuscript reports the successful generation of a non-human primate marmoset model of OTOF-related deafness. After gene therapy was performed in mice models for Otof-related deafness, clinical trials in humans have been done using AAV gene replacement. The transition from mice to humans has been rapid, with a need for exploring safety, efficacy and longevity in an intermediate model. Hence the authors created a CRISPR marmoset with an OTOF knock-out, replicating OTOF auditory synaptopathy. This CRISPR/Cas9-based editing strategy resulted in biallelic knockout animals that exhibit profound hearing loss.

In the introduction, the authors described the marmoset and its advantages as a non-human primate model (NHP). Moreover, they stated the ethical justification since hearing impairment is common and reduces quality of life. These marmosets will be relevant for explosring open questions regarding gene therapy treatment, including capsid-promotor combinations, an immunomodulation protocol and the consequences of treating both ears.

Guide RNAs were used to generate the editing of exon14 of the OTOF gene, with the modification of using 4 guides to avoid indels. A thorough description was provided regarding the creation of the marmoset knock-outs. Auditory synaptopathy was detected by using ABR and DPOAE. No otoferlin was detected in cochleas derived from one of the knock-out marmosets. No evidence was found for evidence for mosaicism, a common issue with marmosets.

We thank this reviewer for the comments and advice to further improve our manuscript. We have completely overhauled the MS following the reviewer's advice, and performed additional experiments and analyses, which further strengthened our manuscript. Below is our point-by-point response to the reviewer's concerns and comments.

Comments for improving the manuscript:

1. When does auditory function emerge in marmosets? Does it occur prenatally in the uterus as in humans, or postnatally as observed in mice? It would be worth adding these points to the introduction.

This has been done. The following text was added: "This is reflected also in a more similar development, anatomy and physiology[5]. For example, humans and NHPs start hearing in utero (onset of hearing in marmosets likely in last month before birth[6]), while rodents show a postnatal hearing onset. This is particularly relevant in the context of gene therapy studies, where postnatal intervention could meet a structurally intact sensory organ in rodents, while degeneration e.g. of dysfunctional hair cells might limit the feasibility of gene therapy in NHPs and humans[7]."

2. What is the similarity of the human and marmoset OTOF human orthologs? Please add this information.

Human canonical OTOF and the long marmoset OTOF isoform (UniProt F7IDY5) are both 1997 aa long. The functionally critical domain architecture (six C2 domains, C-terminal TM helix) is conserved across mammals, and the sequences of human and marmoset OTOF are over 90% identical, even more similar in the C2 domain cores, and almost completely conserved in the TM region[8].

3. The number of live births was quite low. Is this standard for marmoset or might there be an effect by OTOF on development?

In general, the rate of cleavage, progression, and implantation of embryos after interventions such as injection of CRISPR reagents is expected to be lower than with untreated, intact embryos, especially in marmoset, which exhibit species-specific peculiarities of the zona pellucida, ooplasm, and oolemma.

We achieved a major gain in efficiency after optimization of CRISPR activity, injection timing and technique, and optimization of embryo transfer including more efficient synchronisation between the embryo recipient and the developmental stage of the in-vitro-produced embryo. The two genetically modified animals were obtained only after optimization of the injection mixture and the injection stage. We report all results, including those from the initial optimization experiments. Such data are rarely included in final manuscripts, but we consider them essential to prevent unnecessary trials by other groups and, hence and foremost, to reduce animal use in future studies.

After optimization of the injection mixture and stage, we performed five embryo transfers with a total of 15 embryos. Three animals were born (implantation rate 20%; 3/15), of which one was genetically modified. This corresponds to a rate of 20% genetically modified animals per transfer (1/5) and 6.7% per embryo transferred (1/15). Compared with outcomes reported by other groups working on genetic editing in nonhuman primates (reviewed in ref. 1), our results are comparable to or exceed most published values.

In a subsequent experimental phase, we adopted a new anesthesia regimen and, in some cases, transferred frozen–thawed embryos. The impact of each factor on embryo production and implantation will be analyzed in future work and reported separately. Overall, during this phase the yield of genetically modified offspring declined (see Supplementary Table 1) but remained within the range reported for nonhuman primates, supporting the promise of the proposed approach.

For clarity, the outcomes obtained after protocol optimization are summarized in Supplementary Table 1.
Moreover, in response to the reviewer's comment, we added the following statement to the section of the discussion that deals with limitations of our study:

"Only two *OTOF*-KO animals were obtained from 81 embryos transferred in this present study. We achieved a major gain in efficiency from learnings such as on the failure of grafting genotyped embryos and the success with increased CRISPR activity. Our most efficient protocol resulted in rates of 20% genetically modified animals per transfer and of ~7% per transferred embryo (Supplementary Table 1), which is comparable to or exceeds most published values[1]. Future work will generate further animals using the optimized protocol and by cross-breeding the existing *OTOF*-KOs with wildtype marmosets. Parallel efforts of generating *OTOF*-mutant macaques from a spontaneously arisen mutation will help serving the urgent needs described above[2]. This notwithstanding, the generation times will remain a limiting determinant for the availability of the NHP models of DFNB9."

4. The genotypes of two offspring are described. What is the genotype of the third live born animal (line 232)? It's not clear who animal 5 and 6 are relative to the 3 offspring that survived.

We are sorry for the confusion caused. Two of the eight offspring, which had a wild-type genotype died during birth or soon thereafter.

5. As the vocalization behavior was performed at the age of 2 to 4 weeks, and ABR at 6 months, it would be better to describe the results in chronological order.

Thank you for this valuable suggestion, which we thoroughly considered. In conclusion, we would like to refrain from changing the order, so that the deafness phenotype is reported first as this is critical for motivating, presenting, and discussing the vocalization phenotype.

6. At what age was the immunostaining performed? Were the images taken under the same conditions? It seems like there is a residual expression of OTOF, particularly in the left ear of the cochlea shown.

We suspect that the overall higher fluorescence in the otoferlin channel reflects an elevated background signal upon treatment of the left cochlea. We had dosed the animal with *OTOF* dual-AAV in a procedure described in ref. [9], except for not including kanamycin. As mentioned in the MS, the animal had to be euthanized 3 days after surgery due to a complication during anaesthesia. From our experience and that in the field, the time from dosing does not suffice for sufficient transgene expression, which is confirmed by the immunolabeling for the N-terminal alpha tag of otoferlin (Fig. 1 of the letter). Moreover, in response to this comment, we analyzed the immunofluorescence intensity of the otoferlin channel in regions covering inner hair cells and inner border cells in which a transgenic otoferlin expression is highly unlikely (even with broad-acting promotors[10]). We conclude that the observed signal is most likely unspecific and does not represent transgenic otoferlin. We consider it most likely to represent a signal related to the application procedure and not to AAV transduction.

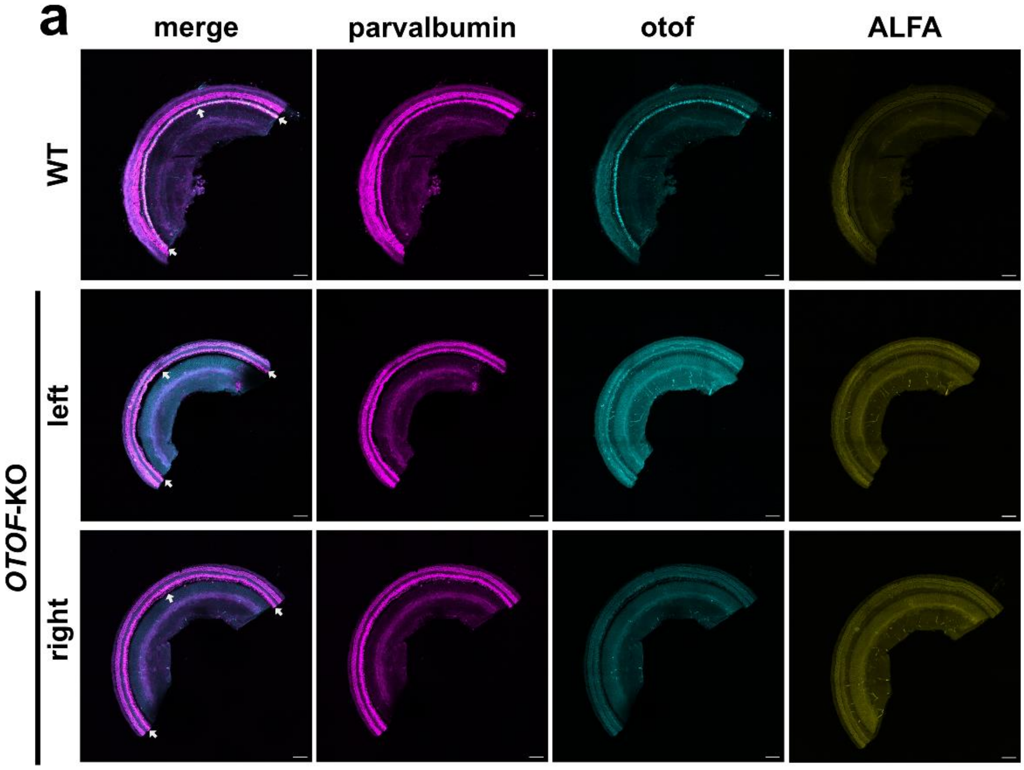

Figure 1 for letter: Immunofluorescence of the organ of Corti of animal 5 (same images as in figure 6 for parvalbumin and otoferlin). Immunofluorescence for the ALFA-Tag present at the C-terminus of the *OTOF*-transgene is depicted in yellow. No specific staining (above the background fluorescence level visible also in the non-treated control animal) was detectable. Scale bar 100µm

7. Did you check the hearing of the marmosets earlier prior to 6 months?

No, this was not analyzed earlier. There is very few experience in anesthesia of very young marmosets. In order to reduce experimental risk for the animals, we planned the experiments for the age of 6

months, where routine ID tag implantation occurs under general anesthesia. For this reason, our approved animal protocol does not allow any testing under anesthesia at an age < 6 months.

Reviewer #4 (Remarks to the Author):

We would like to thank the reviewer for the comments and advice to further improve our manuscript. We have completely overhauled the MS following the reviewer's advice, performed additional experiments and analyses, which further strengthened our manuscript. Below is our point-per-point response to the reviewer concerns and comments.

The field of genetic forms of deafness has known considerable advances in the past 30 years. Since the characterization of the first deafness gene in the 1990s, there are currently more than 150 deafness genes that have been shown to cause isolated deafness and for which the underlying pathophysiological mechanisms have been or are being characterized. Since the 2010s, numerous preclinical attempts have shown that in several models, a substantial recovery of hearing can be achieved. In 2019, complete recovery of auditory thresholds after gene therapy could be demonstrated in mutant mice for the Otof genetic form. In 2024, several consortia around the world have injected the first vectors in patients and analysis of the degree of hearing recovery and its maintenance are under evaluation. The article by Tobias Kahland et al. reports the generation of marmoset monkeys with a non-mosaic disruption of the OTOF gene as a model of human deafness. The creation of a non human primate preclinical model is of importance because it will increase the safety of future generations of vectors rather than translating findings directly from mice to patients. The article is generally well written, clearly exposing the stakes of their work for the auditory field. It describes the challenges encountered to generate a stable model, characterizes the auditory phenotype of the OTOF model, and the nature of their vocalizations. Although this work is very important in terms of impact, a proof of principle on hearing restoration would have been a real plus, even if one has to admit that the long generation time of marmoset means that this work is likely to take several more years.

We would like thank the reviewer for the fair assessment of the challenges associated with working with NHPs. We concur with the conclusion "that a proof of principle on hearing restoration would have been a real plus" which, unfortunately, we have not yet managed to achieve despite >5 years of work on the project. We have now included a discussion of these important points in the section "Value of OTOF-KO marmosets for future work and limitations of the present study".

I have intermediate/minor issues that can in theory be addressed by the authors and are presented by order of appearance in the article :

-Introduction, line 65. The authors claim « some 1/1 human :mouse orthologs have developed new temporal and spatial expression trajectories…. ». Could the authors give precise examples of their claim ? It is generally considered that mice replicate very well the pathophysiology of deafness in humans with very rare exceptions.

The reviewer correctly notes that mice generally replicate the pathophysiology of human hereditary deafness. Indeed, for the great majority of known deafness genes, mouse knockouts or mutants show hearing loss similar to humans[11–13]. However, there are rare but important exceptions where a gene variant causing deafness in humans fails to produce a hearing-impaired phenotype in mice. In these cases, the mouse "equivalent" mutation does not result in hearing loss or causes only mild phenotypic changes, rendering the disease mechanisms difficult to study in mice. Recent research has identified several such one-to-one orthologs and has traced these phenotypic discrepancies to differences in genes expression patterns (spatial and/or temporal) between rodents and primates[14] (Hosoya et al.,

2016). Below we refer to specific examples provided by Hosoya et al., 2016, to substantiate the claim that some 1:1 human–mouse orthologs have diverged in expression and function:

- CRYM (μ-crystallin) – Mutations in *CRYM* cause progressive, adult-onset hearing loss in humans (down-sloping audiogram)[15]. However, *Crym*-knockout mice do not exhibit any hearing impairment[16]. A key difference is that in mice the Crym protein is only weakly expressed in the cochlea (largely confined to the lateral wall fibrocytes and spiral limbus) and is absent from the sensory hair cells[14]. In primates, by contrast, CRYM is broadly expressed in the inner ear – including the hair cells of the organ of Corti and surrounding supporting cells. Thus, the human inner ear relies on CRYM in cell types critical for hearing, whereas the mouse cochlea does not, which likely explains why loss of CRYM is deleterious in humans but benign in mice[14].
- GJB3 (Connexin 31) – *GJB3* encodes a gap-junction protein, and dominant mutations in human *GJB3* cause nonsyndromic hearing loss (DFNA2B/DFNB91) by disrupting cochlear cell–cell communication. Mice lacking *Gjb3* (Cx31), however, have normal hearing under laboratory conditions[17]. One reason is that the function of Cx31 in the mouse cochlea is minor and partially redundant. In mice, Cx31 is expressed only in a subset of supporting cells of the lateral wall (spiral ligament fibrocytes) and spiral limbus, and not in the organ of Corti sensory epithelium[14]. In the primate cochlea, by contrast, Cx31 is expressed more widely – for example, in the spiral ligament (type I fibrocytes), basal cells of the stria vascularis, Reissner's membrane, and supporting cells of the organ of Corti[14]. The broader expression of Cx31 in primates is likely the reason why human *GJB3* mutations can disrupt cochlear electrogenic homeostasis and mechanotransduction more severely than *Gjb3* mutations in mice. In short, the limited Cx31 expression and compensation by other connexins, like Cx26, allows *Gjb3*-mutant mice to retain hearing, whereas humans with *GJB3* mutations develop hearing loss[14] .
- GRHL2 (Grainyhead-like 2) – Heterozygous mutations in the transcription factor encoding *GRHL2* gene cause DFNA28, an autosomal dominant progressive hearing loss in humans[8], and SNPs in GRHL2 have been associated with age-related hearing loss[19]. Mice, however, do not recapitulate this phenotype. *Grhl2*$^{+/-}$ (haploinsufficient) mice have essentially normal hearing, and inner-ear specific knockouts have only subtle effects on cochlear function in experimental settings. Notably, the spatial *GRHL2* expression in the cochlea differs between species. In mouse cochlea, *Grhl2* is strongly expressed in the stria vascularis and limited supporting cell domains, but not in hair cells. In the marmoset cochlea, GRHL2 protein is present in a much broader set of cells – including the spiral ligament fibrocytes, the inner and outer hair cells, their surrounding supporting cells, and spiral ganglion neurons[14]. The absence of Grhl2 in critical sensory cells of the mouse ear (and potential developmental compensation in mice) are the likely reasons why mouse mutants do not show deafness, whereas humans with GRHL2 mutations lose hearing due to dysfunction in the primate-specific expression domains.
- DFNA5 (GSDME) – *DFNA5* is an example of a human deafness gene[20] with a unique pathogenic mechanism. In humans, certain dominant mutations in *DFNA5* (*GSDME*) cause late-onset, progressive hearing loss, probably by creating a truncated gasdermin protein that triggers cell death in cochlear tissues[21]. A knockout of *Gsdme* in mice, on the other hand, has no effect on hearing[22], consistent with the notion that *DFNA5* mutations harm hearing via a toxic gain of function rather than loss of function. In any case, the *DFNA5* expression pattern in the ear also differs between species. In mice, *Gsdme* expression is thought to be largely restricted to the stria vascularis (based on transcriptomics data), whereas in the primate inner ear *DFNA5/GSDME* is broadly expressed – detected not only in the stria vascularis, but also in spiral ligament fibrocytes, inner and outer hair cells, supporting cells, and cochlear neurons[14]. This wider primate expression pattern can explain why human *DFNA5* mutations likely affect many cochlear cell types, whereas a mouse *Gsdme* defect (especially if only a null allele) might be more easily compensated or inconsequential in cells that do not normally rely on GSDME.

- ATP6V1B1 – Recessive mutations in *ATP6V1B1* (encoding the B1 subunit of vacuolar H^+-ATPase) cause distal renal tubular acidosis with sensorineural hearing loss in humans[23]. Mice lacking *Atp6v1b1* show the expected kidney phenotype (metabolic acidosis), but surprisingly do not become deaf – their inner ear development and auditory thresholds remain near-normal[24]. A likely explanation is, again, an inter-species difference in gene expression. Mouse Atp6v1b1 is expressed only in a narrow region of the cochlea (the spiral limbus adjacent to the sensory epithelium). In the common marmoset cochlea, by contrast, ATP6V1B1 is found throughout the lateral wall (spiral ligament fibrocytes), in the spiral limbus, in the hair cells and supporting cells of the organ of Corti, and in the auditory neurons[14]. The human inner ear appears to depend on V-ATPase function in many cell types (endolymph pH homeostasis, etc.), whereas the mouse cochlea may utilize the B2 subunit (ATP6V1B2, which is partly redundant) for these purposes. Thus, losing *ATP6V1B1* impairs hearing in humans, but not in mice, due to different expression patterns and compensation in the cochlea.

Each of the cases above exemplifies how a 1:1 orthologous gene can acquire a new temporal and spatial expression pattern in one lineage, leading to a divergent phenotype when that gene is disrupted. In the five examples (*CRYM*, *GJB3*, *GRHL2*, *DFNA5/GSDME*, *ATP6V1B1*), mutations in the corresponding genes are all known to cause hearing loss in humans, yet the corresponding mouse variants (despite high sequence homology) do not cause comparable hearing loss. Researchers have directly attributed this to differences in gene regulation. The *spatial expression patterns* of these deafness genes in the cochlea are markedly different between rodents and primates[14]. In primates (including humans) the genes tend to be more widely expressed in more cell types or at different developmental stages than in mice, so the human mutations disrupt the auditory system in ways that a mouse mutation does not[14]. In other words *“the discrepancy of gene expression between rodents and primates accounts for the phenotypic difference”* in these models[14].

In response to the reviewer’s comment, we now added the following sentence after the quoted phrase:

“For example, while many forms of monogenic human deafness, including *OTOF*-related auditory synaptopathy, can be modelled in mice, there are documented exceptions for deafness genes *GJB3, CRYM, GRHL2, DFNA5,* and *ATP6B1*[1].”

-introduction line76. The claim « Clearly, a knowledge gap remains regarding the efficacy, specificity and longevity of gene therapy » is surprising coming from a group that contributes or has contributed to the otoferlin gene therapy and taking into account the fact that clinical trials are ongoing in patients. The authors should specify precisely based on articles which aspects need to be further examined carefully and why mice did not answer these questions. Otherwise, this sentence sounds as if enormous risks have been taken by translating findings in mice directly to patients. To the best of my knowledge, although an OTOF NHP model did not exist before, preclinical trias have tested toxicity and expression maintenance in NHPs.

We rephrased the indicated passage in response to this comment: “Further work, ideally including studies in an NHP model, is required to evaluate the efficacy, specificity, and longevity of the gene therapy[1]. For example, the question as to whether expression of transgenic otoferlin in the *OTOF*-deficient primate cochlea is limited to inner hair cells (IHCs, i.e. target-cell specific expression) can only be addressed by post-mortem histology.”

-introduction , line 126. For the role of otoferlin, the authors must cite original papers. A key paper by

Pangrsic et al, 2010 (PMID: 20562868) should be cited and the review is anterior to at least one other key paper like in 2017 (Michalski et al., PMID: 29111973). Together with these two papers, the original paper by Roux et al, 2006 should be recited.

This has been rectified. Some of the indicated references had indeed been cited, but we now introduce them early on.

-Results line 156 : What is special about exon 14 ? Could the authors mention to which otoferlin domain does it correspond and why the choice of that particular exon?

This has been rectified. We found this strategy to successfully delete otoferlin expression in mice. In response to the comment we added this information: "Hence, four sgRNAs (Table 1) were selected using the guide RNA selection tool CRISPOR[25,26] to target exon 14 of the marmoset *OTOF* gene, following a successful knockout strategy employed in mice[27,28]."

-Results : Have the authors tried to determine which Otof isoforms are expressed in the marmoset ? Several Otof isoforms have been reported in humans and mice. There are probably interspecies differences. It would be informative to know if the authors could establish whether there are several OTOF isoforms in marmosets.

The suggested analysis would be of great interest, indeed: To date, data availability is still limited in the field. To our knowledge, no inner *C. jacchus* ear transcriptome dataset is available in the gEAR database or in literature. Exon 14 is present in all seven transcript variants (X1 to X7) predicted for *C. jacchus* at NCBI. The same applies to the four published and nine predicted mouse variants. In contrast, among the five known human Otof transcription variants, exon 14 is only included in variants 1 and 5. Thus, the reviewer's point is well taken and we aim to develop a method for marmoset scRNAseq protocol that will be of great use for our ongoing and future projects.

-Results, line 335. Although I am a non specialist of this field, I respectfully disagree with the word « Intriguingly ». No other species comes close to humans in terms of language. It is probably the major specificity that differentiates us from other species. There has been several papers on mouse call in the past and the same result was found. There are no major differences in the mouse vocalizations between hearing and deaf mice. The article would benefit from a discussion careflully comparing their results with similar studies in deaf mouse models. See for example : PMID: 23536072, PMID: 25062471

In response to the reviewer's comment we revised the MS accordingly: "Taken together, *OTOF*-KO animals were as vocally active as age-matched wild-type controls and produced the full vocal repertoire of infant marmosets. This is reminiscent of related analyses of deaf *Otof* KO mice[29] and other deaf mouse mutants[30]."

-Figure 3 : Marmoset WT ABRs seem to have 3 waves. However, in the supplementary figure, the authors label Waves 1 to 5. Could the authors label the waves in their main figure and give wave details in terms of spieces differences between marmoset, humans and mice.

In marmosets, ABR waveforms are highly variable between animals and strongly depend on – in comparison to mouse – the spatial configuration of recording electrode configuration. Earlier work by others has also demonstrated that different waves are dependent on sound level and can merge (e.g. Harada & Tokuriki, 1997; doi: 10.1016/s0168-5597(96)96015-3). We chose the Vertex to ipsilateral mastoid configuration as in most cases waves II, III and V are visible (Harada & Tokuriki, 1997). Further,

it should be noted that in general the amplitude of marmoset ABR is substantially smaller than in mice but comparable to human clinical ABR.

-Figure 5 : Figure 5 could benefit from bigger insets on IHCs. Why is there so much background in the OTOF KO marmoset compared to the WT control. Haven't the authors used the same settings in both conditions ? If not, this should be detailed.

This has been rectified.
We suspect that the overall higher fluorescence in the otoferlin channel reflects an elevated background signal upon treatment of the left cochlea. We had dosed the animal with *OTOF* dual-AAV in a procedure described in ref. [9], except for not including kanamycin. As mentioned in the MS, the animal had to be euthanized 3 days after surgery due to a complication during anaesthesia. From our experience and that in the field, the time from dosing does not suffice for sufficient transgene expression, which is confirmed by the immunolabeling for the N-terminal alpha tag of otoferlin (Fig. 1 of the letter). Moreover, in response to this comment, we analyzed the immunofluorescence intensity of the otoferlin channel in regions covering inner hair cells and inner boarder cells in which a transgenic otoferlin expression is highly unlikely (even with broad-acting promotors[10]). We conclude that the observed signal is most likely unspecific and does not represent transgenic otoferlin. We consider it most likely to represent a signal related to the application procedure and not to AAV transduction.

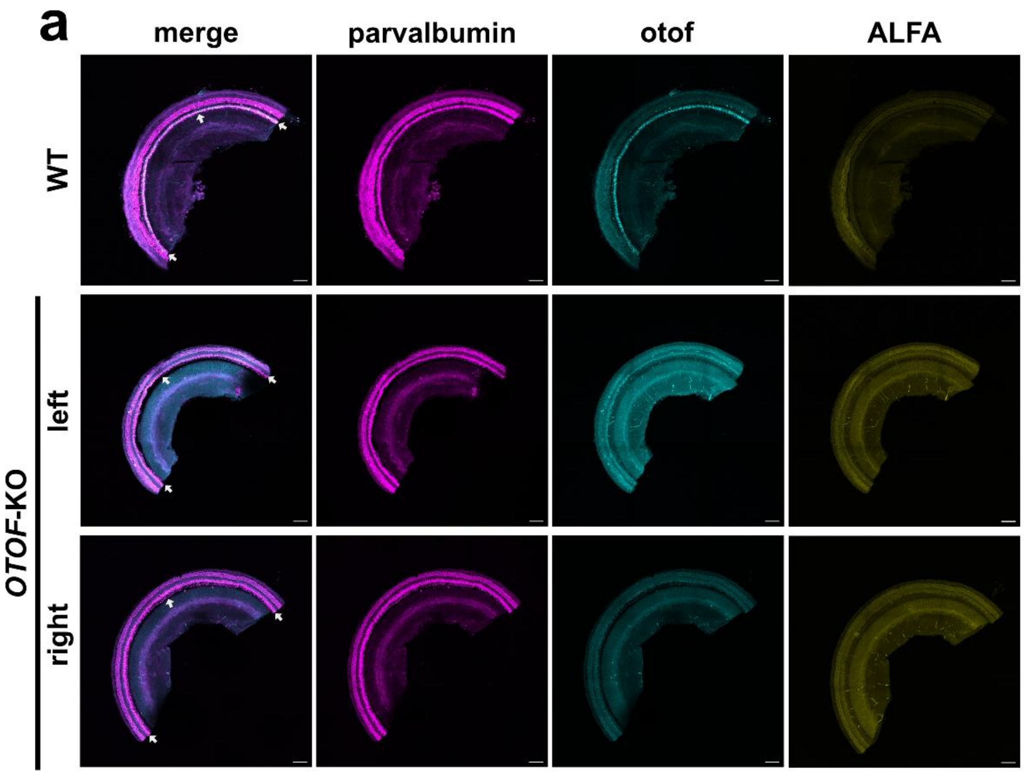

Figure 1 for letter: Immunofluorescence of the organ of Corti of animal 5 (same images as in Figure 6 for parvalbumin and otoferlin. Immunofluorescence for the ALFA-Tag present at the C-terminus of the *OTOF*-transgene is depicted in yellow. No specific staining (above the background fluorescence level visible also in the non-treated control animal) was detectable. Scale bar 100µm

-Discussion paragraph line 423-437. This paragraph is unclear. The authors did not find major differences in their call analysis but cite other articles that found differences in other experimental

settings ; Could the author carefully report differences and comonalities with their study. Have the same developmental periods been compared ? how long were the parental deprivations etc…

In our original submission, we focussed on an early postnatal timepoint and reported a rather coarse analysis in the to introduce the *OTOF*-KO marmosets in general. It is our conviction that a detailed analysis of the acoustic structure is highly interesting, warranted but better suited for a separate manuscript with more data and animals.
We agree with the reviewer that as originally written the MS might be misleading in suggesting no difference in vocal behaviour between KO and WT animals. This interpretation is certainly too strong. Consequently, we have now significantly expanded this analysis and added a new figure 5 where we analysed the maximum call durations for different call types and find that KO animals display shorter vocalizations both in an early (week 2-4) and later (week 18 – 20) time period. While KO animals show increased call durations as expected from a maturation driven process, their call durations do not catch up with WT animals. These data suggest alternative developmental trajectories in WT and KO animals.

-Discussion lines 450-454. The manuscript discussion should more indicate in much more details the reasons why a marmoset model is important for gene therapy trials and for cochlear implants.

This has been rectified in the discussion section "Value of OTOF-KO marmosets for future work and limitations of the present study":

"Here, *OTOF*-KO NHP models could play a critical role in improving *OTOF*-related gene therapy before moving to clinical trials in humans complementing the highly relevant work in mouse mutants. models will allow for extended studies on specificity, efficacy, and longevity of the functional restoration upon gene therapy. For example, otoferlin levels in IHCs and target-cell specificity of transgenic otoferlin expression can now be assessed for different capsid-promotor combinations by post-mortem histology of the treated *OTOF*-KO marmoset cochlea. This will allow to correlate auditory function following *OTOF*-related gene therapy as determined at the physiological and behavioral levels with postmortem analysis of otoferlin expression in IHCs, thereby facilitating the optimization of dosing, vectors, promotors, transgene, and AAV administration. Moreover, NHP models will support late-preclinical studies on the dependence of the functional outcome on time point of intervention, AAV-dose, presence of neutralizing antibodies, and immunomodulation. In addition, *OTOF*-KO marmosets could be useful for the development of next generation cochlear implants. Further, this model uniquely enables the exploration of how auditory feedback shapes vocal development, thereby deepening our understanding of basic neuroscience and driving translational clinical research into auditory disorders. We conclude that the key role of appropriate NHP models of genetic deafness for late-preclinical testing of novel therapies offset the challenges such as slow generation time, risk of off-target effects of CRISPR, potential immune responses to Cas9, high costs and ethical concerns regarding disease modeling in NHP."

-Discussion. I have not found any mention or comparison with other NHP models of deafness like for Usher syndrome. See PMID: 35710827

We thank the reviewer for this comment, now cite the corresponding study, and compare it to ours.

-Suppl Fig. 4. I am not sure to understand properly Suppl figure 4. It is somewhat misleading and gives the wrong impression of the importance of the unexplained waves in animal 6. These waves were obtained at important sound levels compared to wild-type controls. Therefore using a comparison based on hearing threshold gives these waves more unnecessary importance than needed. For

instance, in Fig.1C, the panel gives the impression KO waves are 4 times bigger than in the WT just because there is a larger working range in WT mice. The authors should at least show what happens at higher levels than +30dB above threshold in WT monkeys. In panel « a », the cmparison of a 80 dB signal in WT versus a 100 dB signal in KO is also misleading. Between 80 and 100 dB, there is roughly a 100 fold difference in sound intensity…so putting in parallel these 2 signals that have similar amplitude are misleading. They should put in parallel the KO signal at 80 dB for example.

We thank the reviewer for this important comment, which helps to provide a well-balanced presentation of the data. We aim to report the unexpected finding of an ABR-like response for a specific tone burst frequency (2 kHz) at levels ≥100 dB (SPL) and, hence, the efforts in the original submission to present the some of the data for dB (Hearing Level). We concur with the reviewer that this can be misleading and, therefore, have overhauled Fig. S4.

1. Our protocol first tests click ABR responses. If no click response is seen at all, the pure tone ABR is measured at 100 dB SPL and multiple frequencies. Here, as no click response was observed but a hint for a response at 100 dB SPL for 2 kHz, we measured 2 kHz at 90, 100 and 110 dB SPL. At 90 dB no response was observed. Therefore, no recordings were attempted at lower sound levels. For ethical reasons we did not record ABR traces of WT marmosets for levels >80 dB (SPL).
2. We replotted previous panels b-e for dB (SPL) and added an analysis of the putative response in relationship to trials with stimulation in phase and antiphase.

We have also improved the presentation and discussion of this unexpected finding:

Results:
"In contrast, no waves were observed for clicks even up to a sound level of 100 dB (SPL peak equivalent) ~~for clicks~~ (Fig. 3a) in *OTOF*-KO animals. Unexpectedly, for stimulation with pure tones, an isolated ABR-like response in one ear following stimulation with 2 kHz tone bursts was observed starting at 100 dB (SPL). This response was observed for 2 kHz tone bursts regardless of their phase, indicating that it is unlikely to reflect a microphone potential of hair cells, and showed peak 1 and 2 latencies compatible with those of ABR waves I and II at 80 dB (SPL) in WT marmosets (Suppl. Fig. 4). In no other cases, an ABR was observed (Fig. 3b)."

Discussion:
"We currently do not know the precise mechanism underlying the isolated ABR-like response in one ear following stimulation with 2 kHz tone bursts at levels ≥100 dB (SPL). It phenomenon does not seem to reflect a microphone potential of hair cells. We currently cannot safely distinguish between the possibility of residual sound encoding in the absence of otoferlin, e.g. due to low-level genetic mosaicism (i.e. few WT IHCs, the second *OTOF* KO is to be treated with *OTOF* gene therapy) and a somatosensory or vestibular system origin of the response. Specifically, vestibular nerve compound action potentials with short latencies have been reported for *Otof* KO mice[1]."

Minor:

-Abstract, line 42. The authors should tell why preclinical optimization remains invaluable to establish OTOF-gene therapy.

This has been rectified in the discussion section "Value of *OTOF*-KO marmosets for future work and limitations of the present study".

-Results line 308 : Typo on « A repeated measures ANOVA with within subject… » ?

This has been rephrased.

-line 653. There is a typo « a94% »

This has been rectified.

- I am uncertain there is a Supplementary figure 2 legend on genotyping results

This has been rectified.

-line 43. Typo at « Fig (XB) » ?

This has been rectified.

References

1. Schmidt, J. K., Reynolds, M. R., Golos, T. G. & Slukvin, I. I. CRISPR/Cas9 genome editing to create nonhuman primate models for studying stem cell therapies for HIV infection. *Retrovirology* **19**, 17 (2022).
2. Brigande, J. V. *et al.* Initial Characterization of a Naturally-Arising Otoferlin Frameshift Mutation in the Rhesus Macaque. in (Anaheim, 2024).
3. Doench, J. G. *et al.* Optimized sgRNA design to maximize activity and minimize off-target effects of CRISPR-Cas9. *Nat Biotechnol* **34**, 184–191 (2016).
4. Tycko, J. *et al.* Mitigation of off-target toxicity in CRISPR-Cas9 screens for essential non-coding elements. *Nat Commun* **10**, 4063 (2019).
5. Vallender, E. J. *et al.* Nonhuman primate genetic models for the study of rare diseases. *Orphanet Journal of Rare Diseases* **18**, 20 (2023).
6. Hosoya, M. *et al.* Development of the stria vascularis in the common marmoset, a primate model. *Sci Rep* **12**, 19811 (2022).

7. Moser, T., Chen, H., Kusch, K., Behr, R. & Vona, B. Gene therapy for deafness: are we there now? *EMBO Mol Med* **16**, 675–677 (2024).
8. Leclère, J.-C. & Dulon, D. Otoferlin as a multirole Ca2+ signaling protein: from inner ear synapses to cancer pathways. *Front Cell Neurosci* **17**, 1197611 (2023).
9. Alekseev, A. *et al.* Efficient and sustained optogenetic control of sensory and cardiac systems. *Nat. Biomed. Eng* 1–16 (2025) doi:10.1038/s41551-025-01461-1.
10. Al-Moyed, H. *et al.* A dual-AAV approach restores fast exocytosis and partially rescues auditory function in deaf otoferlin knock-out mice. *EMBO Mol Med* **11**, e9396 (2019).
11. Steel, K. P. & Kros, C. J. A genetic approach to understanding auditory function. *Nat. Genet.* **27**, 143–149 (2001).
12. Cacheiro, P. *et al.* Human and mouse essentiality screens as a resource for disease gene discovery. *Nature Communications* **11**, 655 (2020).
13. Petit, C., Bonnet, C. & Safieddine, S. Deafness: from genetic architecture to gene therapy. *Nat Rev Genet* (2023) doi:10.1038/s41576-023-00597-7.
14. Hosoya, M., Fujioka, M., Ogawa, K. & Okano, H. Distinct Expression Patterns Of Causative Genes Responsible For Hereditary Progressive Hearing Loss In Non-Human Primate Cochlea. *Scientific Reports* **6**, (2016).
15. Abe, S. *et al.* Identification of *CRYM* as a Candidate Responsible for Nonsyndromic Deafness, through cDNA Microarray Analysis of Human Cochlear and Vestibular Tissues*. *The American Journal of Human Genetics* **72**, 73–82 (2003).
16. Suzuki, S. *et al.* μ-Crystallin as an Intracellular 3,5,3′-Triiodothyronine Holder in Vivo. *Mol Endocrinol* **21**, 885–894 (2007).
17. Plum, A. *et al.* Connexin31-Deficiency in Mice Causes Transient Placental Dysmorphogenesis but Does Not Impair Hearing and Skin Differentiation. *Developmental Biology* **231**, 334–347 (2001).
18. Peters, L. M. *et al.* Mutation of a transcription factor, TFCP2L3, causes progressive autosomal dominant hearing loss, DFNA28. *Hum Mol Genet* **11**, 2877–2885 (2002).

19. Van Laer, L. *et al.* The grainyhead like 2 gene (GRHL2), alias TFCP2L3, is associated with age-related hearing impairment. *Hum Mol Genet* **17**, 159–169 (2008).

20. Laer, L. V. *et al.* Nonsyndromic hearing impairment is associated with a mutation in DFNA5. *Nat Genet* **20**, 194–197 (1998).

21. Xiao, Y. *et al.* Gain-of-function variants in GSDME cause pyroptosis and apoptosis associated with post-lingual hearing loss. *Hum Genet* **143**, 979–993 (2024).

22. Van Laer, L. *et al.* Mice lacking Dfna5 show a diverging number of cochlear fourth row outer hair cells. *Neurobiology of Disease* **19**, 386–399 (2005).

23. Karet, F. E. *et al.* Mutations in the gene encoding B1 subunit of H+-ATPase cause renal tubular acidosis with sensorineural deafness. *Nat Genet* **21**, 84–90 (1999).

24. Dou, H., Finberg, K., Cardell, E. L., Lifton, R. & Choo, D. Mice lacking the B1 subunit of H+-ATPase have normal hearing. *Hearing Research* **180**, 76–84 (2003).

25. Concordet, J.-P. & Haeussler, M. CRISPOR: intuitive guide selection for CRISPR/Cas9 genome editing experiments and screens. *Nucleic Acids Research* **46**, W242–W245 (2018).

26. Haeussler, M. *et al.* Evaluation of off-target and on-target scoring algorithms and integration into the guide RNA selection tool CRISPOR. *Genome Biol* **17**, 148 (2016).

27. Roux, I. *et al.* Otoferlin, Defective in a Human Deafness Form, Is Essential for Exocytosis at the Auditory Ribbon Synapse. *Cell* **127**, 277–289 (2006).

28. Reisinger, E. *et al.* Probing the functional equivalence of otoferlin and synaptotagmin 1 in exocytosis. *J. Neurosci.* **31**, 4886–4895 (2011).

29. Hammerschmidt, K. *et al.* Mice do not require auditory input for the normal development of their ultrasonic vocalizations. *BMC Neurosci* **13**, 40 (2012).

30. Mahrt, E. J., Perkel, D. J., Tong, L., Rubel, E. W. & Portfors, C. V. Engineered deafness reveals that mouse courtship vocalizations do not require auditory experience. *J Neurosci* **33**, 5573–5583 (2013).
